# Highly Permeable and Liquid-Repellent Textiles with Micro-Nano-Networks for Medical and Health Protection

**DOI:** 10.1007/s40820-025-01716-1

**Published:** 2025-04-09

**Authors:** Na Meng, Yuen Hu, Yufei Zhang, Ningbo Cheng, Yanyan Lin, Chengfeng Ding, Qingyu Chen, Shaoju Fu, Zhaoling Li, Xianfeng Wang, Jianyong Yu, Bin Ding

**Affiliations:** 1 State Key Laboratory of Advanced Fiber Materials, College of Textiles, Donghua University, Shanghai, 201620 People’s Republic of China; 2 Innovation Center for Textile Science and Technology, Donghua University, Shanghai, 201620 People’s Republic of China; 3 College of Fashion and Design, Donghua University, Shanghai, 200051 People’s Republic of China

**Keywords:** Non-solvent induced phase separation, Protective textiles, Liquidrepellent, Air permeability, Moisture permeability

## Abstract

Current protective clothing often lacks sufficient comfort to ensure efficient performance of healthcare workers. Developing protective textiles with high air and moisture permeability is a potential and effective solution to discomfort of medical protective clothing. However, realizing the facile production of a protective textile that combines safety and comfort remains a challenge. Herein, we report the fabrication of highly permeable protective textiles (HPPT) with micro/nano-networks, using non-solvent induced phase separation synergistically driven by CaCl_2_ and fluorinated polyurethane, combined with spraying technique. The HPPT demonstrates excellent liquid repellency and comfort, ensuring high safety and a dry microenvironment for the wearer. The textile exhibits not only a high hydrostatic pressure (12.86 kPa) due to its tailored small mean pore size (1.03 μm) and chemical composition, but also demonstrates excellent air permeability (14.24 mm s^−1^) and moisture permeability (7.92 kg m^−2^ d^−1^) owing to the rational combination of small pore size and high porosity (69%). The HPPT offers superior comfort compared to the commercially available protective materials. Additionally, we elucidated a molding mechanism synergistically inducted by diffusion–dissolution-phase separation. This research provides an innovative perspective on enhancing the comfort of medical protective clothing and offers theoretical support for regulating of pore structure during phase separations.

**Supplementary Information:**

The online version contains supplementary material available at 10.1007/s40820-025-01716-1.

## Highlights

- Highly permeable protective textiles (HPPT) with micro/nano-networks were fabricated using a non-solvent induced phase separation, synergistically driven by CaCl_2_ and fluorinated polyurethane, combined with spraying technique.
- The optimized HPPT exhibited excellent liquid repellency and air-moisture permeability properties due to the integration and cooperative drive of the low surface energy and the pore structure of the connectivity network, which can be used as desirable protective materials.
- The formation mechanism of micro/nano-network structure was analyzed by molecular dynamics and observed by dynamic phase transition behavior, which numerically combined with theory illustrate the phase transition mechanism of HPPT.

**Supplementary Information:**

The online version contains supplementary material available at 10.1007/s40820-025-01716-1.

## Graphical abstract

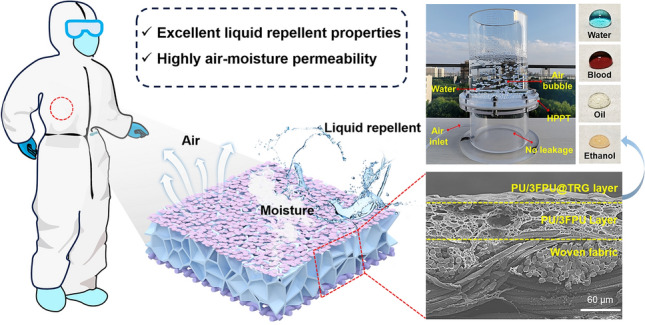

## Introduction

Demand and significance of protective clothing for healthcare workers have significantly increased in recent years since COVID-19 pandemic. These protective textiles are crucial for safeguarding the lives of healthcare workers [1–3]. With ongoing advancements in hygiene standards and quality of life, there is a growing demand for protective materials that offer effective protection while ensuring adequate comfort [4]. Comfort of a textile is triggered by its ability to facilitate effective air and moisture transport [5, 6]. Inefficient comfort characteristics may accumulate heat and moisture, leading to fatigue and restricted wearer performance [7]. Furthermore, it may also result in skin allergies, heat stroke, and even fainting, especially during long shifts that many healthcare workers faced at the height of the COVID-19 pandemic [8–10].

In recent decades, the development of medical protective garments has made great progress in providing enhanced protection from various microorganisms and external fluids, including water and blood [11]. However, relatively little attention has been given to the comfort aspects of protective textiles designed for healthcare workers. Therefore, the critical balance between protective barrier capacity and the wearability of protective membranes—factors that can significantly influence worker performance—remains largely overlooked [12]. Thus, it is essential to design and develop protective textiles capable of providing high comfort without compromising robust protection capability, thereby ensuring a balance between protective barrier capacity and wearing comfort.

Currently, several protective clothing materials are commercially available such as polypropylene melt-blown non-wovens, polyester spun bond non-wovens, PTFE microporous membranes, and cellulose spun bond non-wovens. They are being produced at mass scale and have satisfactory protective properties [13, 14]. Nevertheless, they exhibit poor air and moisture permeability, which is further compromised when subjected to multilayer compounding and reduced pore size, leading to significant discomfort. To address the problem of discomfort, scientists have been making intensive efforts to design protective clothing using traditional woven and non-woven textiles [15]. Various functional materials have been coupled with traditional textiles via coating and laminating techniques to endow them effective protection barrier and comfort for healthcare professionals [16]. Woven fabric after certain surface chemical finishing has been utilized in medical clothing owing to their simple production process, cost-effectiveness, and excellent wearability [17, 18]. Furthermore, multilayer lamination textiles have also been reported to significantly enhance protective performance and multifunctionality [3, 19]. Unfortunately, functional materials for producing these protective textiles still face critical issues such as suboptimal comfort, durability, and cost. Moreover, another effective strategy to prepare protective clothing is to produce barrier membranes on the surface of conventional textiles which may alleviate the problem of comfort while retaining robust protection barrier against the targeted substances [20, 21]. In this regard, non-solvent induced phase separation (NIPS) is believed to be an efficient technology for preparing membranes with tailored asymmetric structure [22, 23]. Shan et al. prepared a aerogel-functionalized thermoplastic polyurethane as waterproof, breathable material using a scalable NIPS strategy [24]. Mandal et al. fabricated porous poly(vinylidene fluoride-co-hexafluoropropene) to create an excellent durability and radiative cooling material by a simple, inexpensive, and scalable phase inversion-based method [25]. Nonetheless, membranes prepared by this method exhibit relatively poor air permeability which can be potentially overcome by the controlled addition of inorganic salts into the precursor solution [26]. Therefore, we believe developing composite structure using conventional textiles coupled with barrier membranes having tailored porous structure and specific surface chemistry would not only offer excellent protection against various kind of liquids as well as exhibit high permeability to balance the protection and comfort characteristics.

Herein, we present a streamlined strategy for manufacturing high permeable protective textiles (HPPT) with micro/nano-networks, using a NIPS, synergistically driven by calcium chloride (CaCl_2_) and fluorinated polyurethane (FPU), combined with spraying technique. Polyurethane (PU) was opted as base material for synthesizing the barrier membrane owing to its soft, breathable, highly elastic, and abrasion resistant nature, which make it suitable material for apparels. CaCl_2_ was used to tailor the porous structure of the membrane, whereas mass fractions of FPU were employed to modulate the phase separation speed. Additionally, commercial hydrophobic agent (TRG) was sprayed to construct low surface energy, enhancing both liquid repellency and hydrostatic pressure of HPPT. We revealed the intrinsic underlying mechanisms utilizing molecular dynamics (MD) simulations and dynamic phase separation behavior analysis. Besides, we also systematically explored the effects of critical factors such as the solubility parameter and diffusion coefficient. The micro/nano-network structure endowed the protective textiles with a balanced combination of small pore sizes, high porosity, excellent permeability, and exceptional surface wettability resistance. In this regard, the obtained HPPT demonstrated good air permeability (14.24 mm s^−1^) and outstanding moisture permeability (7.92 kg m^−2^ d^−1^), along with high hydrostatic pressure (12.86 kPa) and maximum surface wettability resistance (class 5). Notably, the air permeability of HPPT was 8 times higher than that of commercially available high density polyethylene protective membranes (HDPE PM). This research opens new avenues for developing phase separation micro/nano-network textiles that meet the stringent permeability requirements for comfort in personal protection applications.

## Experimental Section

### Materials

Polyurethane (PU, *M*_*w*_ of 50,000 ~ 100,000 g mol^−1^) was acquired from Shanghai Huntsman Polyurethanes Specialties Co., Ltd. Shanghai Taifu Chemical Co., Ltd., China supplied the fluorinated polyurethane (FPU, QF66-2). DMF (AR), anhydrous ethanol (AR, ≥ 99.7%), and CaCl_2_ (AR, 96%) were obtained from Aladdin Biochemical Technology Co., Ltd. A polyethylene glycol terephthalate woven fabric (PET, gram weight containing different mass fractions of FPU of 65 g m^−2^) was supplied by Zhejiang Yisijia Outdoor Equipment Technology Co., Ltd., Jiaxing, China. The fluorinated acrylate three-proof finishing agents (TRG, PH value = 5.0 ~ 6.0, cationic milky white liquid) and the synthetic blood (surface tension of 0.042 ± 0.002 N m^−1^) were brought from Shanghai Xinwu Textile Technology Co., Ltd., China, and Dongguan Chuangfeng Automation Technology Co., China, respectively.

### Fabrication of Uniform Initial Solution

The CaCl_2_ was dissolved in DMF at room temperature with stirring for 3 h, and then, PU and FPU were sequentially added and stirred for 10 h at room temperature to obtain a uniform PU/FPU/CaCl_2_ casting solution. The casting solutions were made by mixing the following amounts of PU, CaCl_2,_ and FPU in 17 mL of DMF: 1.5 g of PU to 1.5 g CaCl_2_ (1:1 PU: CaCl_2_ by mass), 0.2, 0.6, 1, and 1.4 g of FPU (mass fractions of FPU in total solution of 1, 3, 5, and 7 wt%). The obtained uniform casting solutions were abbreviated as PU/1FPU/CaCl_2_, PU/3FPU/CaCl_2_, PU/5FPU/CaCl_2_, and PU/7FPU/CaCl_2_, respectively.

### Fabrication of PU/FPU Membranes

Firstly, the casting solution containing different mass fractions of FPU was spread on a woven fabric substrate (200 mm × 300 mm × 0.14 mm in length, width, and thickness, respectively) with a 9 mL solution volume in the homemade laboratory membrane applicator. The effective membrane size was 16 cm × 25 cm, the casting solution thickness was 250 µm, and the method for determining casting solution thickness was described in Supplementary Methods of Supporting Information (Fig. S1). Afterward, it was immediately submerged in 40 °C water for 2 h to induced phase separation. Subsequently, the membranes were washed in pure water and oven-dried at 60 °C. Finally, PU/FPU membranes were obtained and abbreviated as PU/1FPU, PU/3FPU, PU/5FPU, and PU/7FPU, respectively.

### Fabrication of HPPT

In this embodiment, the PU, FPU, CaCl_2,_ and DMF submerged into the coagulation bath upon submersion, leaving behind a porous PU/FPU framework. Subsequent spraying of the PU/FPU membranes resulted in a highly permeable protective micro/nano-network textile. Obtained textiles were abbreviated as PU/FPU@TRG textiles (i.e., HPPT). Spraying was performed with a gravity-fed spray gun (H2000, Fujiwara Jidori Enterprise Store) with a 1 mm gun bore. The operating air pressure was 4 Bar, the spray distance was fixed at 20 cm, and the gun flow rate was 30 mL min^−1^.

## Results and Discussion

### Design and Preparation of HPPT

**Fig. 1:**
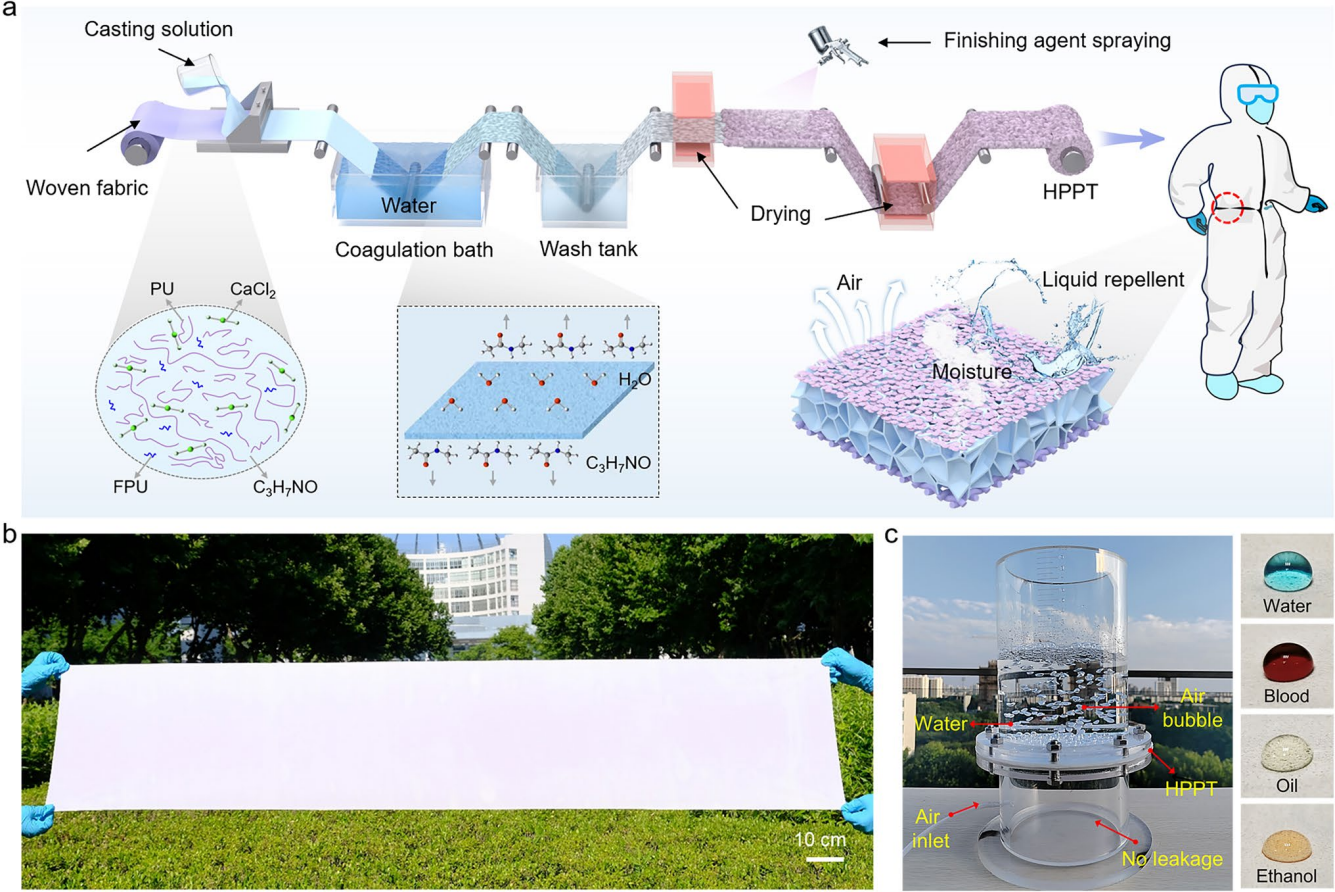
Fabrication scheme and process design of HPPT. **a** Fabrication procedure and application schematic of HPPT with micro/nano-networks. **b** Digital photograph of large-sized HPPT by scalable NIPS technology. **c** Photography demonstrating excellent liquid-repellent and permeability of HPPT

We designed HPPT with micro/nano-networks adopting a NIPS, synergistically driven by CaCl_2_ and FPU, combined with spraying technique, as schematically illustrated in Fig. 1a. A new concept in this work was integrated CaCl_2_ and fluoropolymer during the phase separation process to achieve tailored interconnected porous network, resulting in the formation of highly permeable protective textiles. The prepared HPPT prevents the penetration of external liquids while allowing internal air and moisture to permeate, thereby creating a comfortable microclimate for the wearer. In this work, a homogeneous casting solution composed of PU and FPU (modulators, modifiers, and mediators), CaCl_2_ (pore-forming agent), and DMF (solvent) was evenly dispersed on the woven fabric using doctor blade method. Then, the obtained wet membrane was immediately submerged in 40 °C water (non-solvent) for 2 h to induced phase separation. During this process, water diffused into the initial PU/FPU/CaCl_2_ solution, whereby CaCl_2_ dissolved into the non-solvent, leaving behind a three-dimensional porous PU/FPU scaffold. Subsequently, the membrane was washed with tap water to remove the residual solvent and then dried at 60 °C to obtain the PU/FPU micro/nano-network membrane. To clarify the effect of FPU on the regulation of the phase separation process, we controlled the mass fraction of FPU in the casting solution and developed membranes with tunable micro/nano-networks on the surface (Fig. S2). Then, we investigated the pore size, hydrostatic pressure, air permeability, and moisture permeability of PU/FPU membranes with different FPU mass fractions. As the mass fractions increased, pore size decreased, the hydrostatic pressure increased and permeability decreased (Figs. S3 and S4). These outcomes evidently demonstrate the mass fraction of 3 wt% has achieved a balance between comfort and liquid repellency, and the membrane was abbreviated as PU/3FPU. Additionally, a three-proof finishing agent (TRG) was sprayed onto the membrane surface to reduce surface energy for improving liquid repellency, and the ultimately generated membrane was abbreviated as HPPT.

Remarkably, the HPPT was readily scaled to a large size of 0.4 m × 2 m by applying our unique phase separation technique (Fig. 1b), indicating its potential industrialization in personal protection and healthcare. The balance of protection and excellent air permeability of HPPT is vividly demonstrated in Fig. 1c. The HPPT (sample area of 177 cm^2^) successfully prevented the infiltration of 2 L of water while allowing rapid air through, attributed to the micro/nano-network structure. The corresponding dynamic demonstration of waterproofing and air permeability is shown in Movie S1. Moreover, prepared samples displayed robust repellency to all kinds of liquids (Fig. 1c).

### Morphology and Structure Characterizations of HPPT

**Fig. 2:**
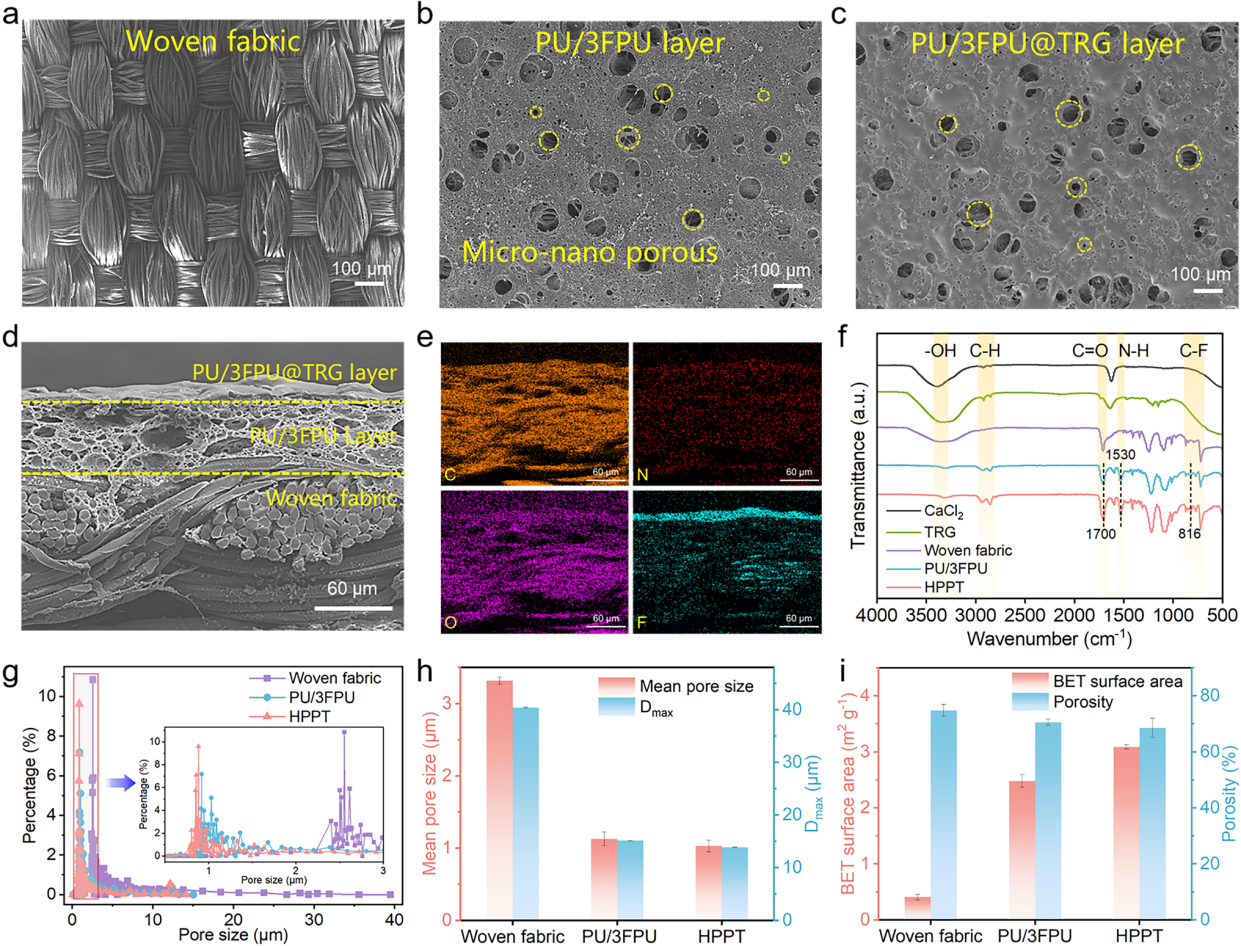
Morphology and structure characterizations of HPPT. **a**–**c** SEM images of woven fabric (**a**), PU/3FPU layer (**b**), PU/3FPU@TRG layer (**c**). **d**, **e** The cross section and corresponding elemental mapping images of HPPT. **f** FTIR spectrum of CaCl_2_, TRG, woven fabric, PU/3FPU, and HPPT. **g** Pore size distribution of woven fabric, PU/3FPU, and HPPT. The inset shows a partial enlargement image of the curves. **h**, **i** Mean pore size and D_max_ (**h**), and porosity and BET surface areas (**i**) of woven fabric, PU/3FPU, and HPPT

To gain insight into the different morphology and structure characterizations of the prepared textiles, samples were subjected to thorough examination via the scanning electron microscopy (SEM), as exhibited in Fig. 2a–c. It is apparent from Fig. 2a, b that the PU/FPU/CaCl_2_ coating significantly altered the smooth surface morphology of the woven fabric, exhibiting rough micro/nano-networks. Moreover, it can be seen from Fig. 2c that there was visible change in pore structure after TRG spray, and density of pore structure increased significantly on the surface of the resultant PU/3FPU@TRG sample. To further confirm the existence of porous structure throughout the fabric, cross section of the fabricated HPPT was carefully analyzed using SEM. It can be noticed from Fig. 2d that interconnected micro/nano-cavities were present throughout the fabric. The presence of pores of difference scale in three layers of the HPPT also confirmed hierarchical porous network and existence of significant number of pathways ensuring robust permeability.

Surface chemistry plays vital role in liquid repellency; therefore, prepared samples were thoroughly investigated via energy-dispersive spectroscopy mapping to comprehend the surface chemical composition of synthesized HPPT (Fig. 2e). The characteristic elements (C, N, O, and F) were uniformly distributed throughout the scanning view, and the accumulation of F element on the surface indicated successful deposition of the TRG. To further assess the chemical composition, HPPT samples were subjected to Fourier-transform infrared spectroscopy (FTIR) analysis. Obtained FTIR spectra exhibited that no typical CaCl_2_ peaks, suggesting its dissolution and removal during phase separation (Fig. 2f). Meanwhile, the C–F stretching vibration peaks at 816 and 1100–1350 cm^−1^ were significantly improved, confirming the presence of enhanced amount of F because of the finishing agent sprayed on the surface of HPPT [27].

It is well established that the liquid repellency and permeability of materials are closely related to pore size and porosity [28]. Therefore, it is vital to thoroughly investigate the porous structure of the textiles. As illustrated in Fig. 2g, the pore size distribution of HPPT was narrower (0.11–14.19 μm) and more regular compared to the original woven fabric, and the pore size majority of HPPT was concentrated at 0.88 μm. Owing to the special micro/nano-networks, the mean pore size and maximum pore size (D_max_) of PU/3FPU and HPPT were significantly smaller than those of the woven fabric (Fig. 2h), with HPPT having a mean pore size of 1.03 µm and a D_max_ of 13.90 µm. As illustrated in Fig. 2i, HPPT showed a larger Brunauer–Emmett–Teller (BET) surface area (3.09 m^2^ g^−1^) than woven fabric (0.41 m^2^ g^−1^). Besides, synthesized HPPT retains significantly high porosity of 69% even after TRG coating. Notably, the presence of tiny pores could effectively block the penetration of external liquids, while the high porosity provides abundant channels for the transport of internal air and moisture [29]. Critically, a large BET surface area would supplement efficiency of moisture transport via rapid adsorption of human moisture [30, 31].

### Mechanism of the Phase Separation Pore Formation

**Fig. 3:**
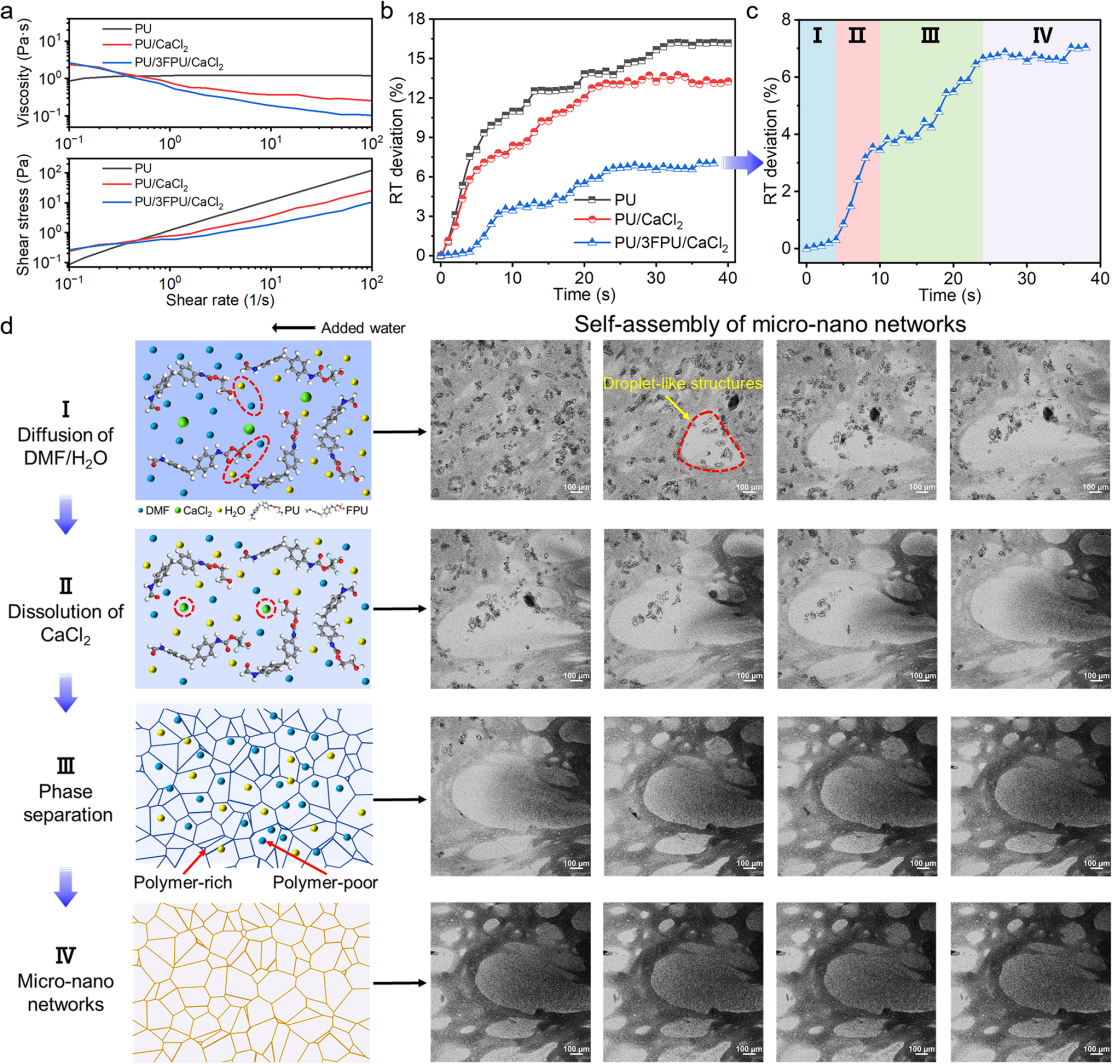
Mechanism of the phase separation pore formation. **a** The viscosity and shear stress of three solution systems of PU, PU/CaCl_2_, and PU/3FPU/CaCl_2_. **b** RT deviation with the time of three solution systems. **c** RT deviation and the four stages of PU/3FPU/CaCl_2_ solution system. **d** Self-assembly process of micro/nano-networks: diffusion of solvent and non-solvent, dissolution of CaCl_2_, phase separation of polymer-poor/polymer-rich phases, and pore formation

Phase separations are a particularly complex physical process involving diffusion, dissolution, and fluid flow [32]. The occurrence of phase separation in solution is influenced by multiple factors, including the concentration and identities of the components, environmental conditions such as temperature, and the type and concentration of salts [33]. Figure 3a demonstrates the viscosity and shear stress as functions of the shear rate for three solution systems (PU, PU/CaCl_2_, and PU/3FPU/CaCl_2_). The PU/3FPU/CaCl_2_ solution displayed extremely low viscosity and the further decreased with increasing shear rate. This is mainly attributed to the addition of the FPU and CaCl_2_, which disrupted the entanglement of PU molecular chains and rearrange the intermolecular interaction forces between the solution molecules. More importantly, the addition of inorganic salt particles orientated the solution along the flow direction and the viscosity decreased [34]. It is well known that the lower viscosity of the polymer solution facilitates the phase separation and enables pores formation [32]. Microimaging, a primary tool for observing liquid–liquid phase separation, plays an indispensable role in comprehending the behavior of polymer, solvent and non-solvent during phase separation process [35]. To delve deeper into the phase separation behavior of the solution systems, a facile method was employed to capture the dynamics of the process (Supplementary Methods, Supporting Information) [36]. The images displaying the dynamic of the three solution systems are displayed in Fig. S5. Interestingly, the addition of CaCl_2_ and FPU not only reduced the solution curing rate but also triggered a staged phase separation in the solution system, which was favorable for the formation of micro/nano-network structures. Figure 3b presents the relative transmittance (RT) deviation curves over time for the three solution systems. The RT deviation of PU/3FPU/CaCl_2_ exhibited relatively slower growth rate compared to the other solutions, indicating a delayed phase separation rate. More importantly, PU/3FPU/CaCl_2_ also showed the lowest RT deviation and highest RT (Fig. S6), suggesting its potential for preparing controlled porous network of cavities.

Further analysis of RT deviation curves for the PU/3FPU/CaCl_2_ solution system revealed that there are roughly four stages of phase separation process (Fig. 3c). The fabrication of the micro/nano-networks depends on the diffusion of solvent and non-solvent, dissolution of CaCl_2_, phase separation of polymer-poor/polymer-rich phases, and pore formation. Figure 3d illustrates the generation of micro/nano-networks via four stage self-assembly process using schematic mechanism (left) and the corresponding evolution of optical morphology of PU/3FPU/CaCl_2_ solution (right). Initially, a water phase (40 °C) was placed on the surface of casting solution phase. Due to temperature disequilibrium between the phases, water diffused across the interface. This diffusion induced directed fluctuations, leading to the formation of droplet-like structures at off-critical and critical compositions (stage I). Further, the diffusion of the aqueous phase soon started dissolving the CaCl_2_, and the casting solution generates more disruption initiated under the competing effects of diffusion and dissolution, i.e., polymer-rich region and polymer-sparse region. The polymer-rich regions appeared in the critical portion and the grayscale of micrographs increased (stage II). Uneven surface concentration due to droplet interactions generated a gradient of surface tension along/near the interface, yielding a hydrodynamic force that drives the solution to undergo two-phase separations, forming polymer-rich and polymer-sparse zones (stage III). Eventually, the grayscale of the microscopic images stabilized, and the micro/nano-networks were shaped (stage IV). In this part, we elucidate in depth the evolution mechanism of micro/nano-networks, providing a theoretical basis for precisely regulating the NIPS.

### Interaction Mechanisms of Casting Solutions

**Fig. 4:**
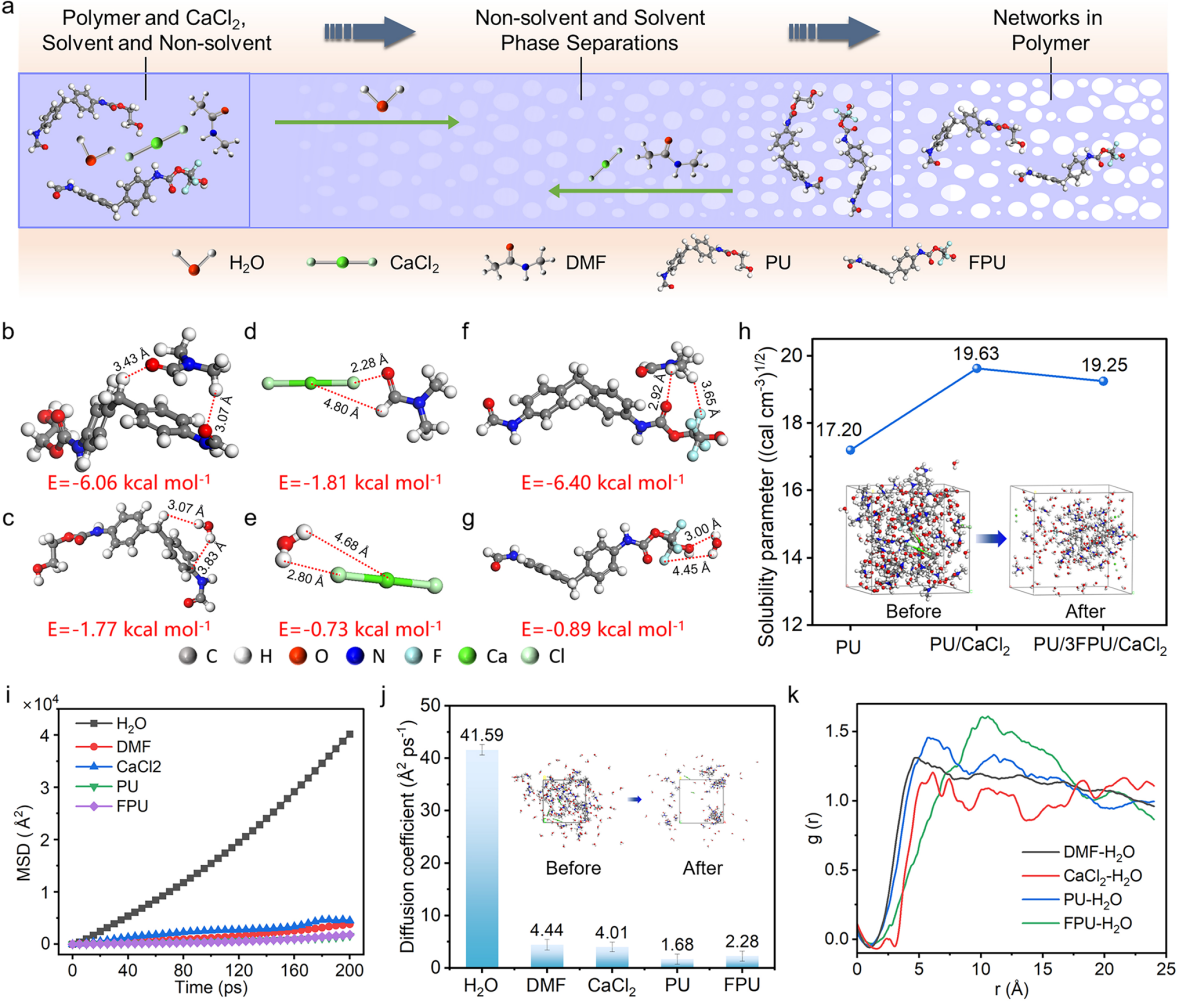
Interaction mechanisms of casting solutions. **a** Schematic of the NIPS process, showing the formation of micro/nano-network textile coating from a solution of DMF, water, and PU/FPU. **b**–**g** Molecular models of PU/DMF (**b**), PU/H_2_O (**c**), CaCl_2_/DMF (**d**), CaCl_2_/H_2_O (**e**), FPU/DMF (**f**), FPU/H_2_O (**g**). **h** Solubility parameter of PU, PU/CaCl_2_, and PU/3FPU/CaCl_2_ in water. The inset in (**h**) shows the model before and after the calculation of the dissolution parameters. **i**–**j** MSD (**i**) and diffusion coefficient (**j**) of water, DMF, CaCl_2_, PU, and FPU in the casting solution. The inset in (**j**) shows the modeled plots of the calculated diffusion coefficient trajectory at 1 and 20 ps. **k** RDF of water with DMF, CaCl_2_, PU, and FPU in the casting solution

Based on the experimental and mechanistic research in the previous section, we summarize the forming process of the PU/FPU/CaCl_2_ micro/nano-network textile in Fig. 4a. To reveal the pore formation mechanism, the process of wet membrane immersion in water was investigated. Given a certain degree of randomness in the porous microstructures produced by the phase separation, statistical characterization becomes increasingly attractive [37, 38]. MD simulations were employed to explain the interaction mechanisms within the casting solution. Detailed MD calculations are provided in the supplementary methods (Supporting Information). To investigate the phase separation behavior, the optimized structures among various molecules were first simulated, and their interaction energies (*E*) were also calculated. *E* is defined as [39, 40]:

1 $$E={E}_{total}-{E}_{frag}^{A}-{E}_{frag}^{B}$$

where ${E}_{total}$ is the total energy of the full complex, and ${E}_{frag}^{A}$ and ${E}_{frag}^{B}$ are the total energies of isolated molecule A and molecule B, respectively. As illustrated in Figs. 4b–g and S7, S8, *E* of FPU/DMF (− 6.40 kcal mol^−1^) was more negative than PU/DMF (− 6.06 kcal mol^−1^) and CaCl_2_/DMF (− 1.81 kcal mol^−1^), while *E* of CaCl_2_/H_2_O (− 0.73 kcal mol^−1^) was more positive than PU/H_2_O (− 1.77 kcal mol^−1^), FPU/H_2_O (− 0.89 kcal mol^−1^), and DMF/H_2_O (− 0.87 kcal mol^−1^). These results indicated that the addition of FPU and CaCl_2_ resulted in a slower phase separation behavior of the precursor solution, consistent with previous findings [29]. The interactions between the dopant solution and non-solvent can significantly influence the polymer behavior and phase separations, as determined by solubility parameters [41, 42]. The solubility parameters of three various solution systems in water are displayed in Fig. 4h. The solubility parameter of the pure PU solution increased from 17.20 (cal cm^−3^)^1/2^ to 19.63 (cal cm^−3^)^1/2^ with the addition of CaCl_2_. However, when both CaCl_2_ and FPU were added, the solubility parameter decreased to 19.25 (cal cm^−3^)^1/2^. These results further verified that FPU effectively slowed down the dissolution of the dopant solution during the phase separation, thus preventing macropores formation [43, 44]. The model before and after the calculation of the dissolution parameters is exhibited in the inset of Fig. 4h.

Beyond the mixture compatibility, the diffusion rate between dopant solution and non-solvent, a principal kinetic aspect, can quantify the phase separation process [45, 46]. Therefore, MD simulations were also employed to further numerically understand the diffusion behavior of molecules within the solution systems. Figure 4i, j shows the mean square displacement (MSD) and diffusion coefficients of the five molecules, in which the MSD of H_2_O molecule was larger than DMF, CaCl_2_, PU, and FPU, indicating that water has superior diffusion properties. The diffusion coefficients for H_2_O, DMF, CaCl_2_, PU, and FPU were calculated as 41.59, 4.44, 4.01, 1.68, and 2.28 Å^2^ ps^−1^, respectively, indicating that the diffusion between the precursor solution and the non-solvent is predominantly governed by the H_2_O molecule. This was attributed to the robust interactions between water and solution molecules due to the different polarities [47, 48]. Apparently, the diffusion between DMF and H_2_O (stage I) occurred preferentially during the phase separation of the PU/FPU/CaCl_2_ solution system was further substantiated by numerical analysis. It is well known that the first peak in the radial distribution function (RDF) corresponds to the first coordination layer around the central molecules, and the first peak multiplied by the average density is the coordination number [49]. Figure 4k shows the RDF of the different molecules with water for the precursor solutions. It was noted that the first peaks for DMF/H_2_O, CaCl_2_/H_2_O, PU/H_2_O, and FPU/H_2_O appeared at 4.71, 5.23, 5.71, and 7.91 Å, respectively, indicating that DMF was closest to the water molecule, followed by CaCl_2_ [50]. These results substantiated the earlier outcomes of dominance of stage II (dissolution of CaCl_2_) and stage III (phase separation of the polymer) in the phase separation.

### Protective Properties and Comfort of HPPT

**Fig. 5:**
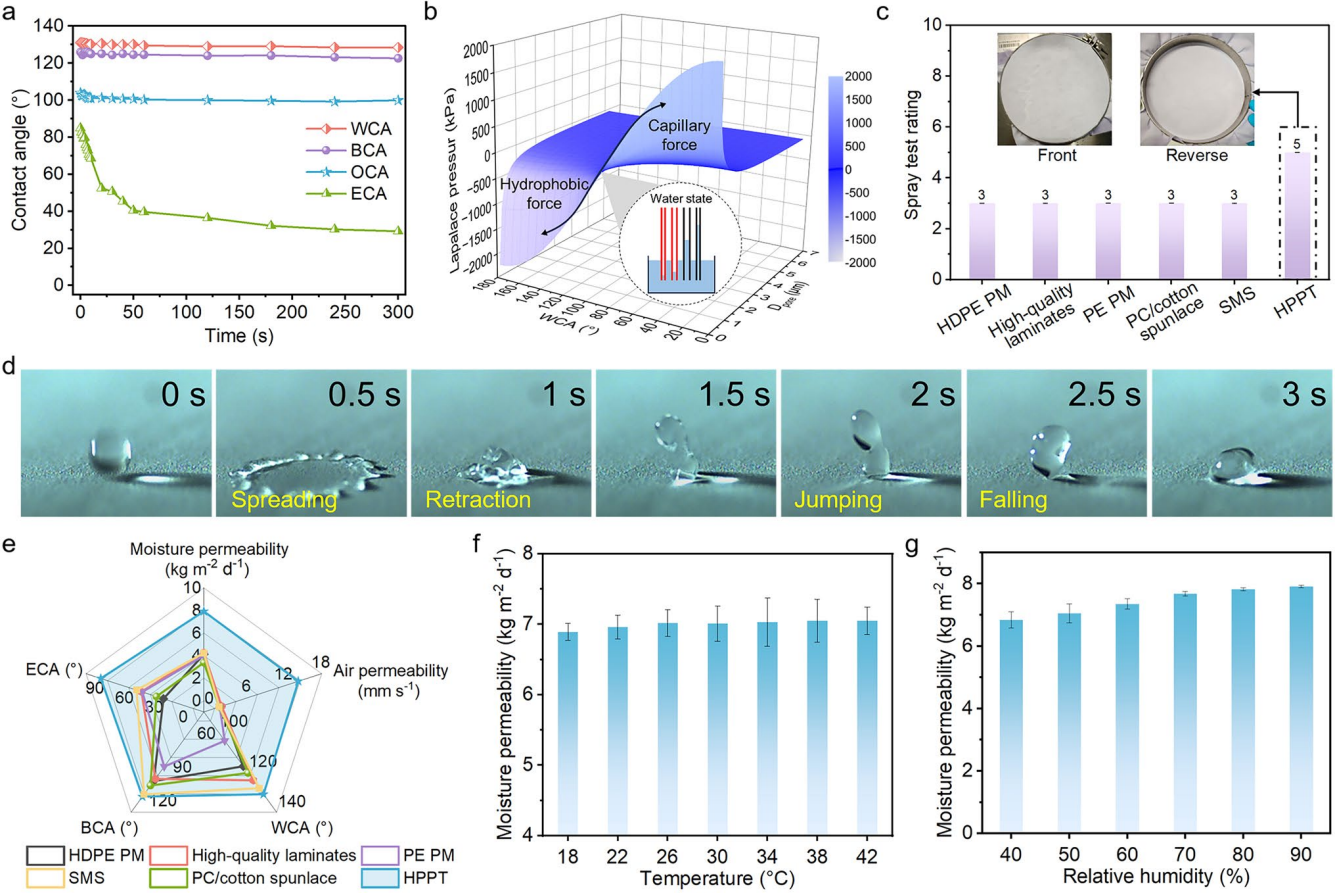
Protective properties and comfort of HPPT. **a** Dynamic behavior WCA, BCA, OCA, and ECA of HPPT over 300 s. **b** Capillary force and hydrophobic force based on Young–Laplace equation in the micro/nano-network textile. **c** Comparison of surface wettability resistance of HPPT with five commercially available materials to dyed water. **d** Snapshots of the impact dynamics for water on HPPT captured by high-speed camera equipment. **e** Comparison of liquid repellent and comfort between the prepared multifunctional HPPT and commercially available protective products. **f**–**g** The moisture permeability of HPPT for (**f**) different temperatures and (**g**) relative humidity

The water wetting behavior of woven fabric, PU/3FPU, and HPPT was investigated to elucidate the modifications in the surface wettability resistance during the HPPT preparation procedure, as displayed in Fig. S9. As expected, when the water droplets were on the HPPT, they could maintain the original shape with a water contact angle (WCA) of 131°. Notably, the hydrophobicity of HPPT remained consistent, as the WCA stayed at 130° even after 300 s. Figure 5a displays the dynamic WCA, blood contact angle (BCA), oil contact angle (OCA), and ethanol contact angle (ECA) of HPPT within 300 s. The HPPT demonstrated excellent WCA (131°), BCA (126°), OCA (104°), and ECA (85°), which can be attributed to its tiny pores and low surface energy (Fig. S10). It is noteworthy that WCA, BCA, and OCA remained essentially unchanged over time, while ECA showed a gradual decrease. These results further confirmed the superior anti-wettability properties of HPPT. Generally, when water droplets are in contact with porous membranes, they enter capillary channels under Laplace pressure. Young–Laplace equation ($P=\frac{4\gamma \bullet \text{cos}\theta }{{D}_{pore}}$) elucidates the magnitude of Laplace pressure, indicating two opposing forces driving water motion (Fig. 5b) [28]. This depends mainly on the pore structure and surface wettability resistance.

Furthermore, we conducted the spray tests and impact dynamics experiments to simulate real scenarios for water interaction with HPPT, as indicated in Fig. 5c, d. When the HPPT surface was sprayed with dyed water, no wetting and adhesion on the surface were observed (inset of Fig. 5c). Thus, the surface wettability resistance of HPPT was determined as class 5 in accordance with ISO 4920:2012(E), level 5 according to GB/T 4745, 100 according to AATCC 22. In contrast, five commercially available materials were assessed at class 3, and the wetting degree on the sample front and reverse sides after spray test is shown in Fig. S11. Strikingly, the water droplet underwent a “spreading-retraction” stage followed by a mild rebound, as demonstrated in Fig. 5d and Movie S2, thus proving its superb liquid repellency of HPPT. The hydrostatic pressures of HPPT and five commercial protective products were contrasted to demonstrate the significant potential for medical applications (Fig. S12). The hydrostatic pressure of HPPT was 12.86 kPa comparable to HDPE PM (12.51 kPa), showcasing its ability to industrialize. To verify the stability of HPPT, the hydrostatic pressure of HPPT under different temperatures was analyzed (Fig. S13). As shown in Fig. S13b, HTTP displays stable resistance against considerably high hydrostatic pressure at a wide range of temperature (25–50 °C). HPPT maintains steady resistance to hydrostatic pressure of 11.69 kPa even at scorching temperature of 50 °C. The antimicrobial performance is one of the important attributes of medical protective clothing. To evaluate the antibacterial effect of HPPT, the zone of inhibition test method is adopted with the bacterial models of *E*. *coli* and *S*. *aureus*. The bacterial suspensions were transferred to a cell culture plate in an agar plate and incubated at 37 °C for 24 h to observe the antibacterial effect. As shown in Fig. S14, it was observed that inhibition ring appeared around HPPTs due to the added bacteriostatic materials during the manufacturing process, which thoroughly proves the excellent antibacterial effect of the HPPT.

Excellent air and moisture permeability is critical for protective garments intended for comfort and long-term wear, which allows effective transfer of sweat vapor outside from the microenvironment between skin and fabric, keeping the skin dry and comfortable [51–53]. Figure 5e presents a radar chart that compares integrated the multidimensional data of commercial textiles with current work. Notably, the HPPT exhibited a fairly higher air permeability (14.24 mm s^−1^) and moisture permeability (7.92 kg m^−2^ d^−1^) compared to its counterparts, which was approximately 8 times (1.78 mm s^−1^) and 2 times (4.11 kg m^−2^ d^−1^) higher than those of HDPE PM. This superior performance was primarily due to the interconnected microchannels in the micro/nano-networks, which facilitated the passage of gas and water molecules [54–56]. Protective clothing with excellent moisture permeability under different temperatures and humidity enhances the comfort and efficiency, which is significant to wearer who work for a long time. Therefore, we assessed the moisture permeability of HPPT under different temperatures and humidity (Fig. 5f, g). With rising temperature and humidity, moisture permeability improves. This phenomenon could be attributed to highly porous structure of HPPT, which enables them to rapidly absorb water molecules from the humidity. Noticeably, the moisture permeability remained consistently high; even when exposed at 42 °C and 50% for 1 h, it remained at 7.05 kg m^−2^ d^−1^. It thoroughly proves the exceptional performance stability of HPPT in extremely humid and hot environments, expanding the range of potential applications.

### Real-Life Applicability of HPPT

**Fig. 6:**
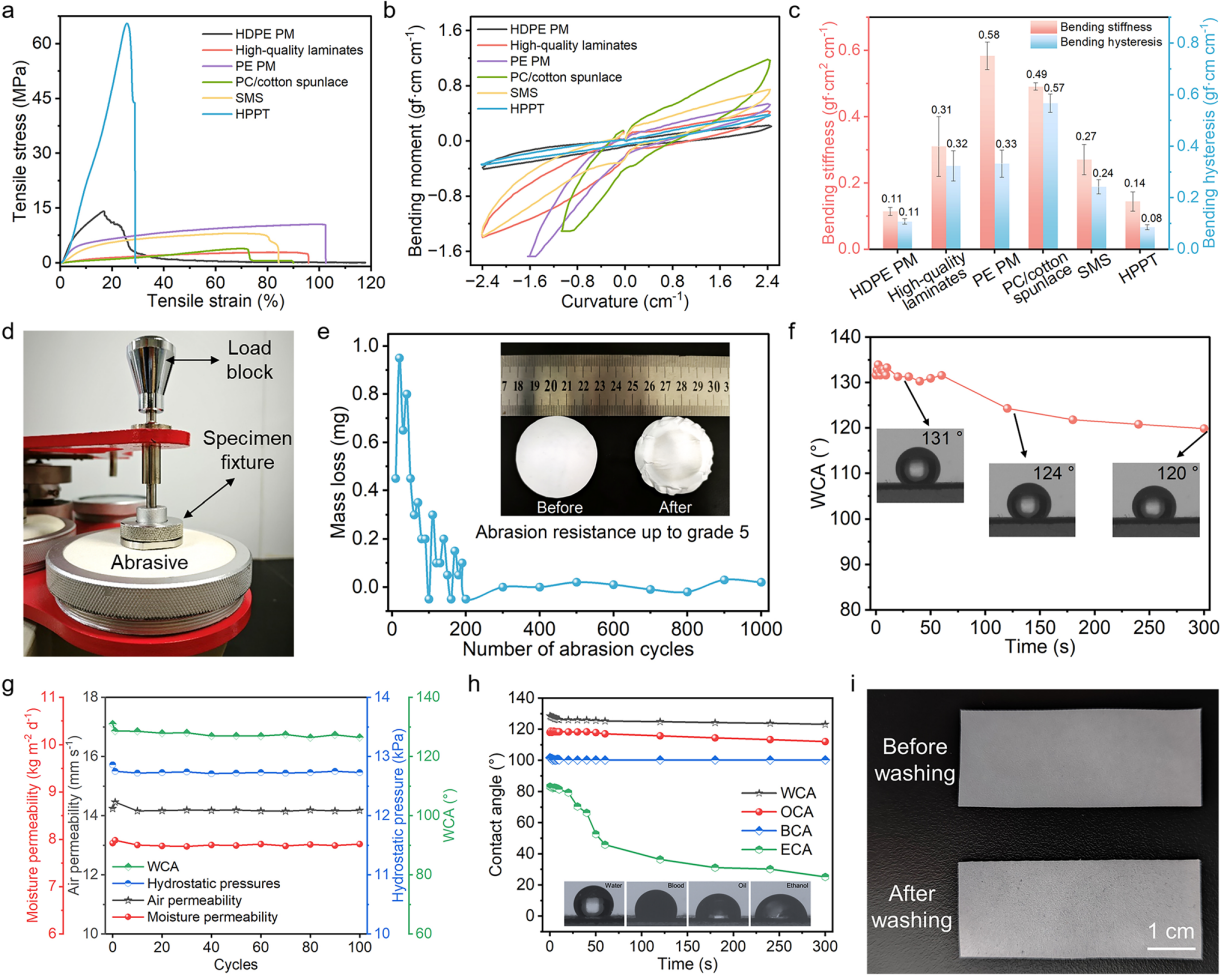
Real-life applicability of HPPT. **a**–**c** Stress–strain curves (**a**), torque–curvature relationship curves (**b**), and bending stiffness and hysteresis (**c**) of HPPT and five commercially available protective materials. **d** Photographs of the abrasion resistance testing apparatus. **e** Sample mass loss variations under various abrasion cycles. The inset in **e** shows the photo-images of the sample before and after 1000 abrasion cycles. **f** The dynamic WCA of HPPT in 300 s after 1000 abrasion cycles. **g** Air permeability, moisture permeability, hydrostatic pressures, and WCA of HPPT as a function of washing cycles. **h** Dynamic WCA, BCA, OCA, and ECA of HPPT in 300 s after 100 cycle washing test. The inset in (**h**) shows the photography of water, blood, oil, and ethanol solutions at first contact with the HPPT. **i** Digital photograph of HPPT before and after 100 cycle washing test

Mechanical characteristics of textiles are believed to be of high significance when it comes to apparels as they tend to determine the durability of the textile. Therefore, to further satisfy long-term and wearable applications, the mechanical properties and abrasion resistance of HPPT were carefully evaluated. Figure 6a presents the stress–strain curves of HPPT and five commercially available protective materials. HPPT demonstrated excellent mechanical properties with a tensile strength of 65.56 MPa, which is ~ 5 times greater than that of HDPE PM (14.07 MPa), one of the best performing commercially available materials. Simultaneously, HPPT exhibited superior flexibility and foldability (Fig. S15). Lower bending moment, bending stiffness, and bending hysteresis indicate better softness and recovery from bending deformation [57]. Using the KES-FB2S bending tester according to GB/T 18318.5-2009, we quantified the bending properties of woven fabric, PU/3FPU, HPPT, and five commercially available protective materials (Figs. S16 and 6b, c). The principle of bending properties test is provided in supplementary methods. It was observed that the bending moment, bending stiffness, and bending hysteresis of HPPT, respectively, were 0.35, 0.14, and 0.08 gf cm cm^−1^, which larger than woven fabric and PU/3FPU, suggesting the bending performance deteriorated after the phase separation coating and spray finishing (Fig. S16). However, HPPT still displayed outstanding bending performance compared to the HDPE PM commercially available protective materials (Fig. 6b, c).

Abrasion resistance is also a crucial parameter for evaluating the wearability and endurance of fabrics [58]. Figure 6d–f displays photographs of the abrasion resistance testing apparatus, sample mass loss variations under various abrasion cycles, and the dynamic WCA of HPPT after 1000 abrasion cycles over 300 s, respectively. The results indicated that the mass loss of HPPT essentially stayed constant as the number of abrasion cycles increased. To clarify the commercial advantages of HPPT in comparison with other commercially available protection materials, the dynamic WCA of five commercial protective materials in 300 s after 1000 abrasion cycles was tested (Fig. S17). It was worthwhile to mention that HPPT maintained superior waterproofing ability after 1000 abrasion cycles. The laundry-proof property of HPPT is a critical parameter for coated textile. To evaluate the effect of the washing cycles on functional characteristics of HPPT, we separately tested the air permeability, moisture permeability, hydrostatic pressures, and WCA of HPPT after different washing cycles (Fig. 6g). Remarkably, the air permeability, moisture permeability, hydrostatic pressure, and WCA of HPPT were 14.46 mm s^−1^, 7.98 kg m^−2^ d^−1^, 12.75 kPa, and 129° after one cycle washing, respectively, indicating negligible change in performance results. Therefore, samples were rigorously washed up to 100 cycles to thoroughly analyze their durability and stability of functional performance. The results further indicate that air and moisture permeability was increased, and the liquid-repellent performance appeared slightly decreased as the cycle of washing continued to increase. This is because TRG layer has minute loss after washing; however, dense porous structure helped the HPPT retain its excellent liquid repellency. Furthermore, TRG degradation probably uncovered more micro- and nano-pores which led to increase the air and moisture permeability. To further validate the stability property of the liquid repellent of HPPT, the dynamic contact angles against various liquids over 300 s were measured (Fig. 6h). It was observed that prepared HPPT exhibited very stable and robust liquid repellency against most liquids even after 100 washing cycles. As illustrated in Fig. 6i, physical appearance of HPPT and its dimensions were still intact after 100 cycle washing test indicating that it has strong laundry-proof property.

To validate the universal applicability and scalability of NIPS technology, synergistically driven by CaCl_2_ and FPU, we demonstrated the feasibility of manufacturing HPPTs based on commercially available woven, non-woven, and knitted fabric (Fig. S18, material parameters in Supporting Information). It was noticed that all the fabrics prepared by this technology showed very promising results, indicating scalability potential of the technology and its feasibility to develop HPPT from a wide range of conventional textiles. More significantly, the high compatibility of our devised process with existing industrial low-cost production processes makes it promising technology to attract the attentions of industry. With the rapid progress and development in electronic technology, computer technology, and smart wearable technology, the flexible protective materials with the ability to monitor health conditions are highly desirable [59, 60]. We also intend to utilize the scientific and technological results of this research to develop bio-based materials with antiviral/antimicrobial films for radiative cooling and electronic textiles, and to expand the applicability of our products to flexible electronic devices and the construction industry.

## Conclusions

In summary, HPPT was successfully developed via a straightforward and rapid strategy combining NIPS and spraying technology for medical and health protection applications. Equipped with a micro/nano-network, the protective textile demonstrated superior air and moisture permeability, as well as excellent liquid repellency. The spraying of the three-proof finishing agent TRG on PU/3FPU resulted in low surface energy and dense pore structure. As a result, HPPT demonstrated remarkable integrated properties, including high WCA (131°), BCA (126°), OCA (104°), and ECA (85°), a hydrostatic pressure of 12.86 kPa, and a tensile strength of 65.56 MPa. Additionally, the connected pores facilitated the transport of air and moisture, ensuring a dry and comfortable microenvironment. HPPT achieved the air and moisture permeability of 14.24 mm s^−1^ and 7.92 kg m^−2^ d^−1^, respectively, which were approximately 8 and 2 times higher than HDPE PM. This work provides novel and valuable insights into improving comfort of protective clothing and encourages the application of HPPT in smart textiles.

## Supplementary Information

Below is the link to the electronic supplementary material.

Supplementary file1 (DOCX 5351 KB)

Supplementary file2 (MP4 4247 KB)

Supplementary file3 (MP4 9190 KB)
