# Supplementary material for: Highly Permeable and Liquid-Repellent Textiles with Micro-Nano-Networks for Medical and Health Protection
Source: Nanomicro Lett. 2025 Apr 9;17:208. doi: 10.1007/s40820-025-01716-1 (PMC11982006; doi:10.1007/s40820-025-01716-1)
Supplement: Supplementary file 1 — Supplementary file1 (DOCX 5351 KB) [file 40820_2025_1716_MOESM1_ESM.docx]

Supporting Information for

**Highly Permeable and Liquid-Repellent Textiles with Micro/Nano-Networks for Medical and Health Protection**

Na Meng^1,2^, Yuen Hu^1^, Yufei Zhang^1^, Ningbo Cheng^3^, Yanyan Lin^1^, Chengfeng Ding^1^, Qingyu Chen^1^, Shaoju Fu^1^, Zhaoling Li^1^, Xianfeng Wang^1,2^*, Jianyong Yu^2^, Bin Ding^2^*

^1^ State Key Laboratory of Advanced Fiber Materials, College of Textiles, Donghua University, Shanghai 201620, P. R. China

^2^ Innovation Center for Textile Science and Technology, Donghua University, Shanghai 201620, P. R. China

^3^ College of Fashion and Design, Donghua University, Shanghai 200051, P. R. China

*Corresponding authors. E-mail: [wxf@dhu.edu.cn](mailto:wxf@dhu.edu.cn) (Xianfeng Wang); [binding@dhu.edu.cn](mailto:binding@dhu.edu.cn) (Bin Ding)

S1 Supplementary Figures


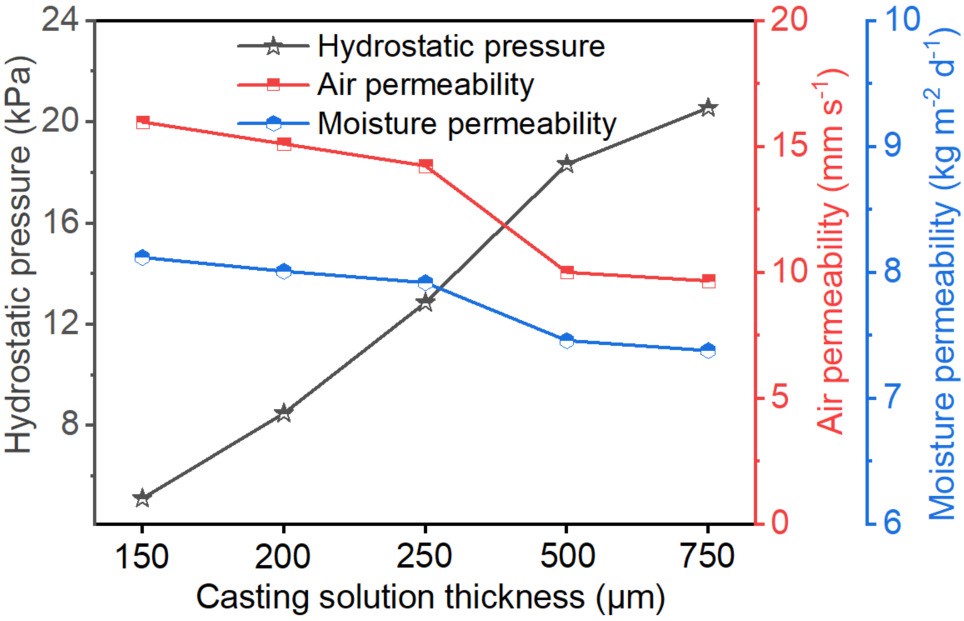


**Fig. S1** The hydrostatic pressure, air permeability and moisture permeability of different casting solution thickness


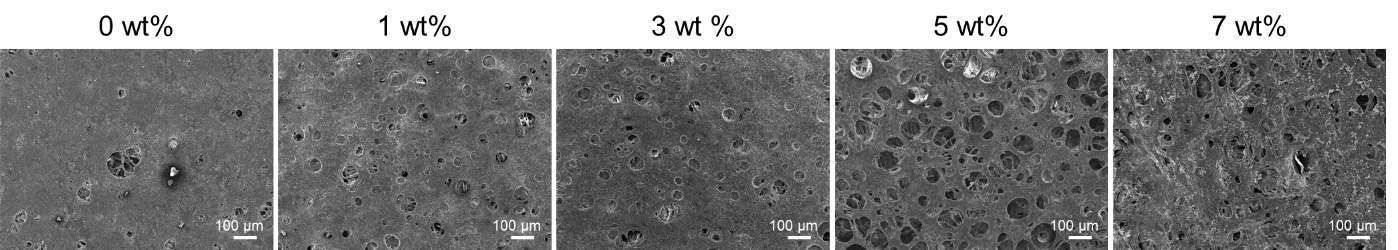


**Fig. S2** SEM images of micro-nano network membranes with different FPU mass fractions


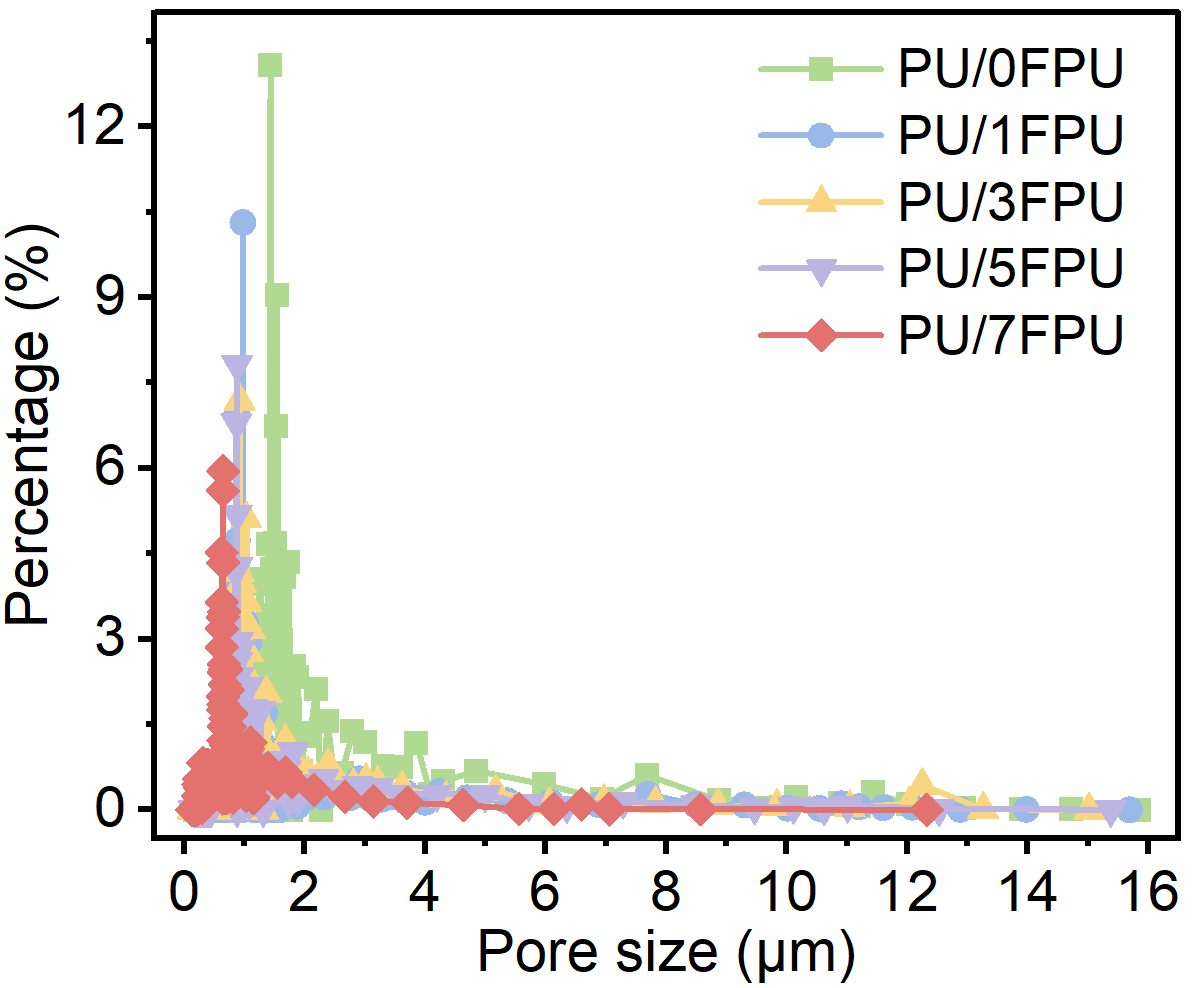


**Fig. S3** The pore size distribution of micro-nano network membranes with different FPU mass fractions


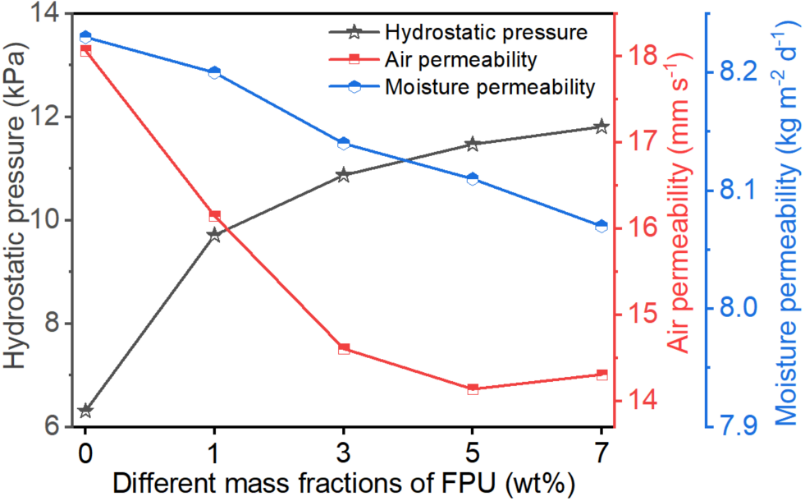


**Fig. S4** Hydrostatic pressure, air permeability, and moisture permeability of micro-nano network membranes with different FPU mass fractions

**
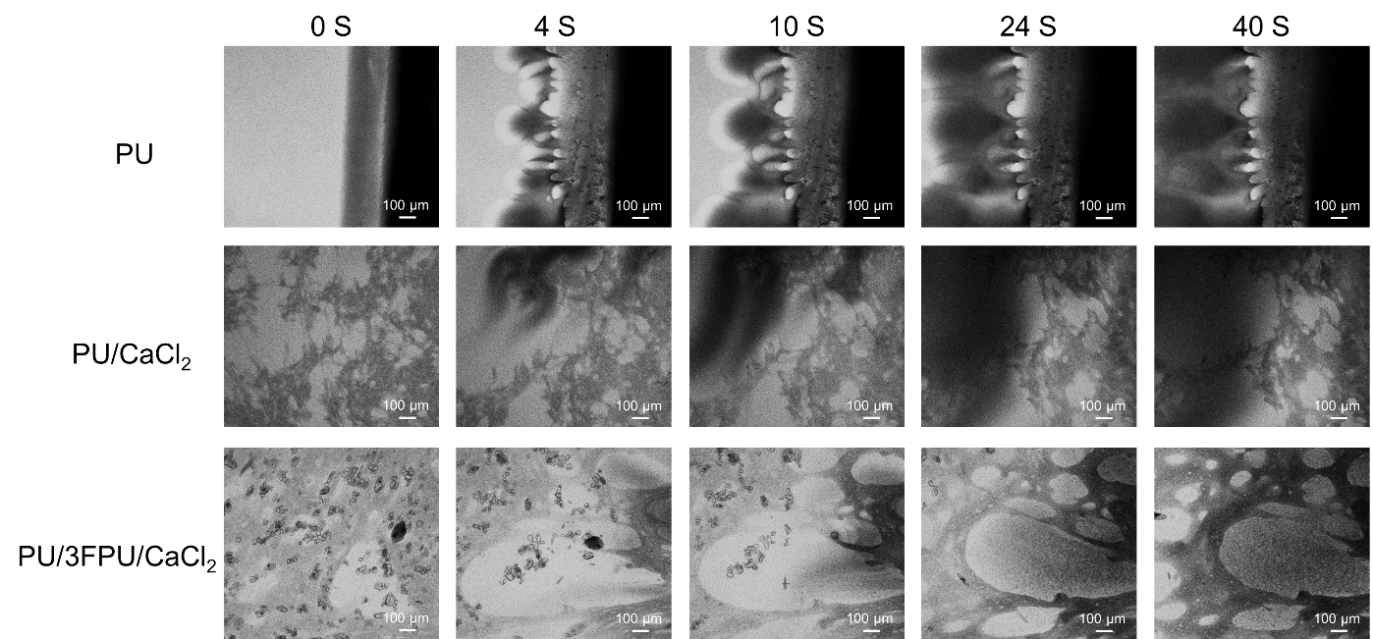
**

**Fig. S5** Dynamic images of the phase inversion behavior on three solutions systems


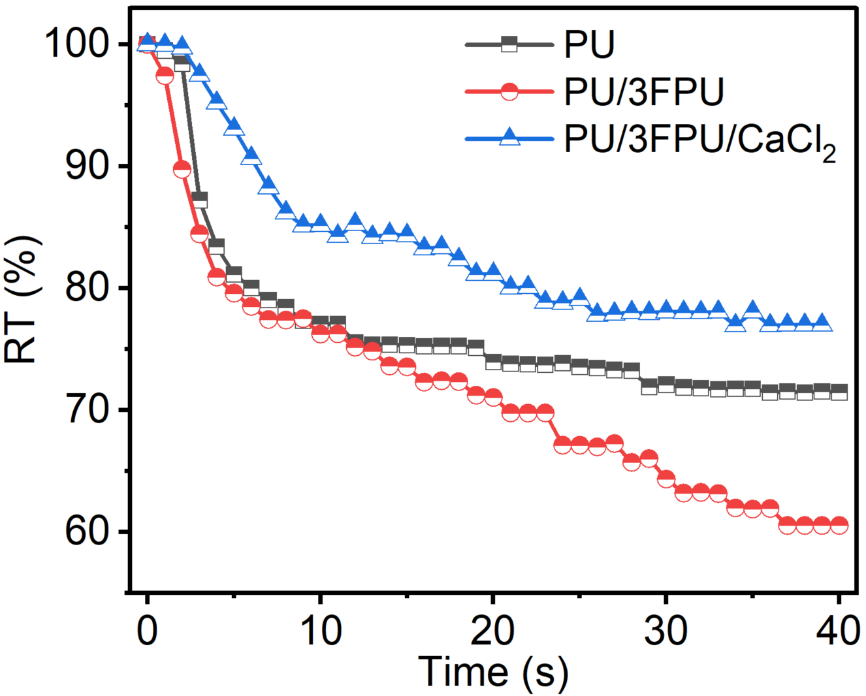


**Fig. S6** RT curves of three solution systems


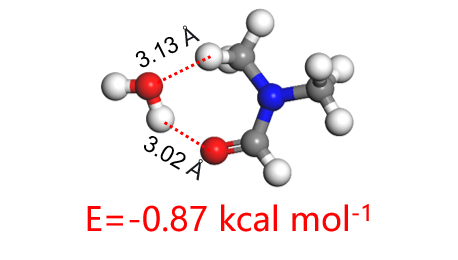


**Fig. S7** Molecular models of DMF/H_2_O


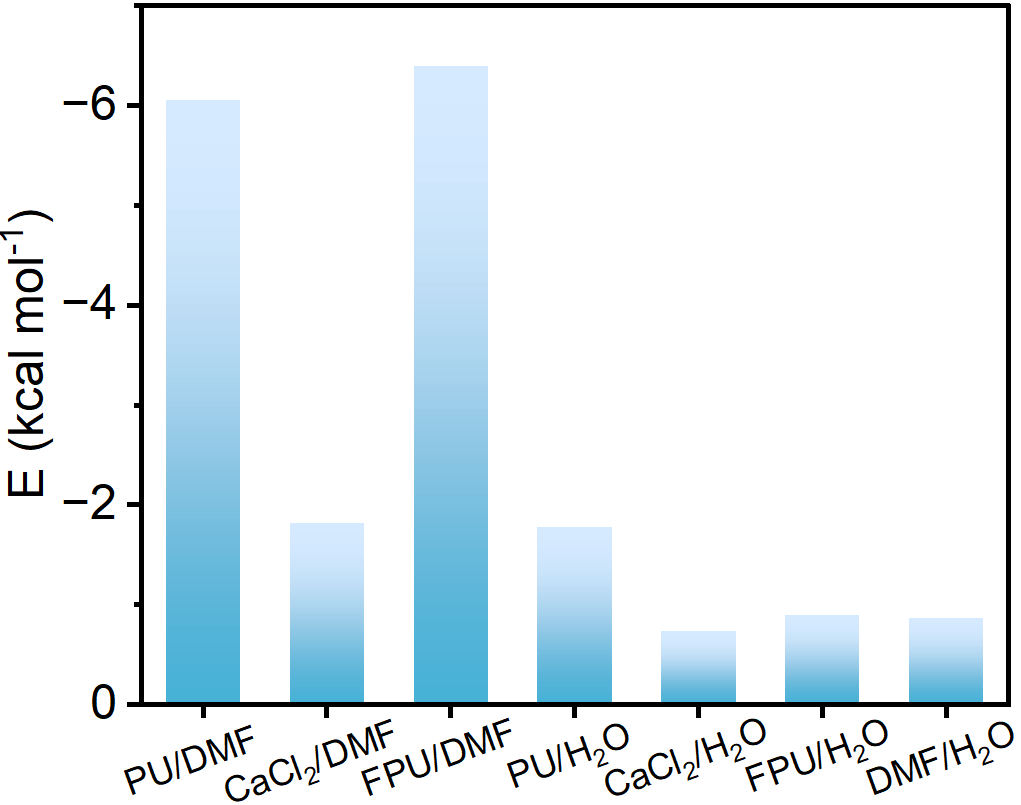


**Fig. S8** Interaction energies of various molecules in the casting solution


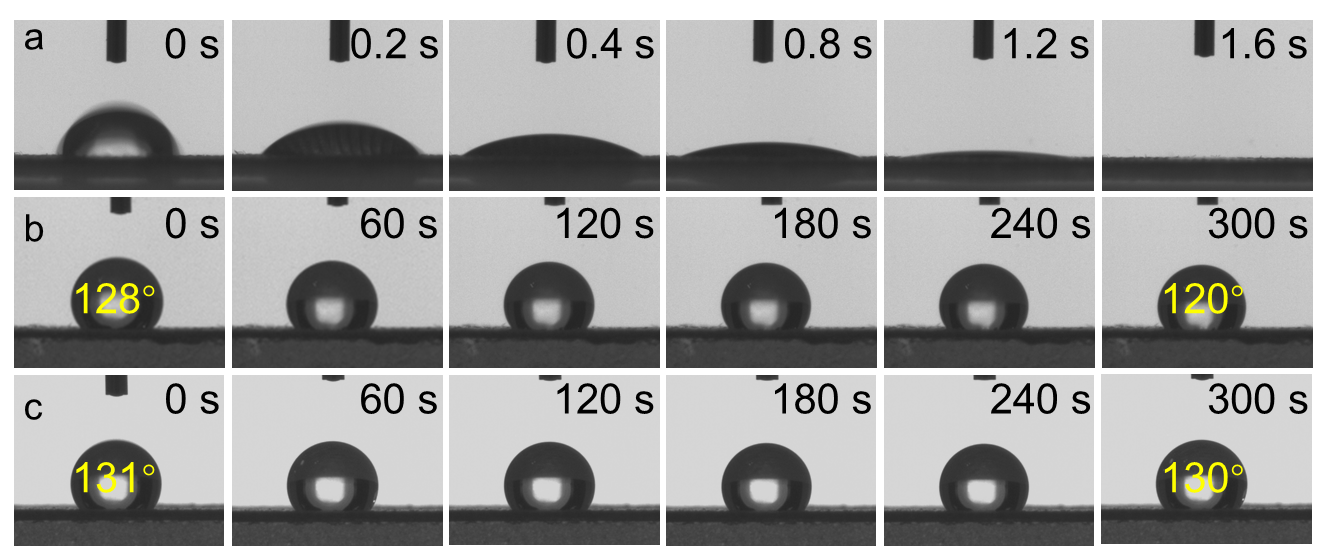


**Fig. S9** The apparent WCA dynamics change with time as water drops on the woven fabric (**a**), PU/3FPU (**b**), and HPPT (**c**)


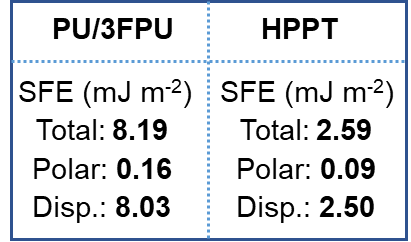


**Fig. S10** Total SFE, polar SFE and disp. SFE of PU/3FPU and HPPT


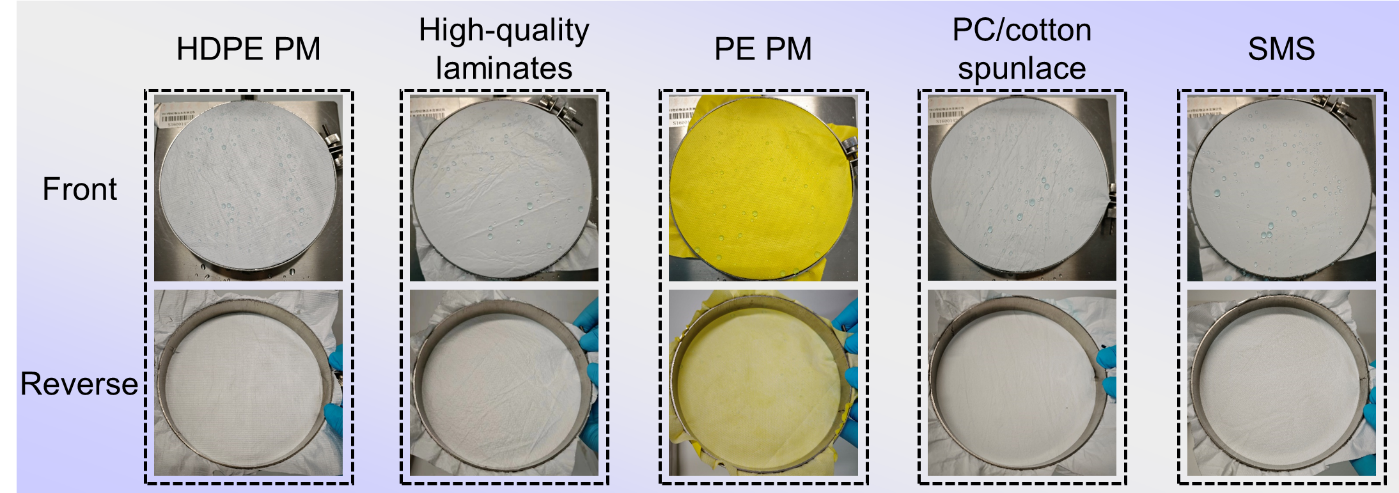


**Fig. S11** The degree of wetting on the front and reverse of the HDPE PM, High-quality laminates membranes (High-quality laminates), Polyethylene protective membranes (PE PM), PC/cotton spunlace non-woven (PC/cotton spunlace), and Spun-bond Melt-blown Spun-bond non-wovens (SMS) at the end of the water spray test, respectively


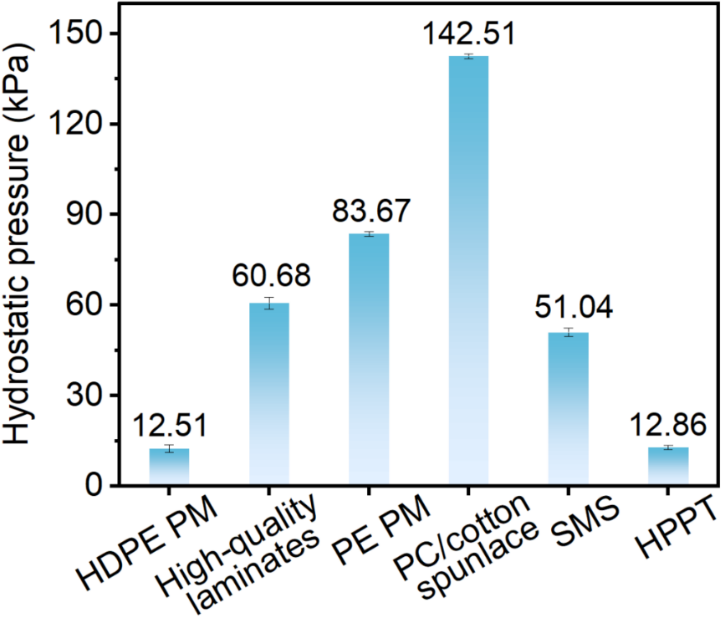


**Fig. S12** The hydrostatic pressures of HPPT and five commercial protective clothing products using laboratory test method


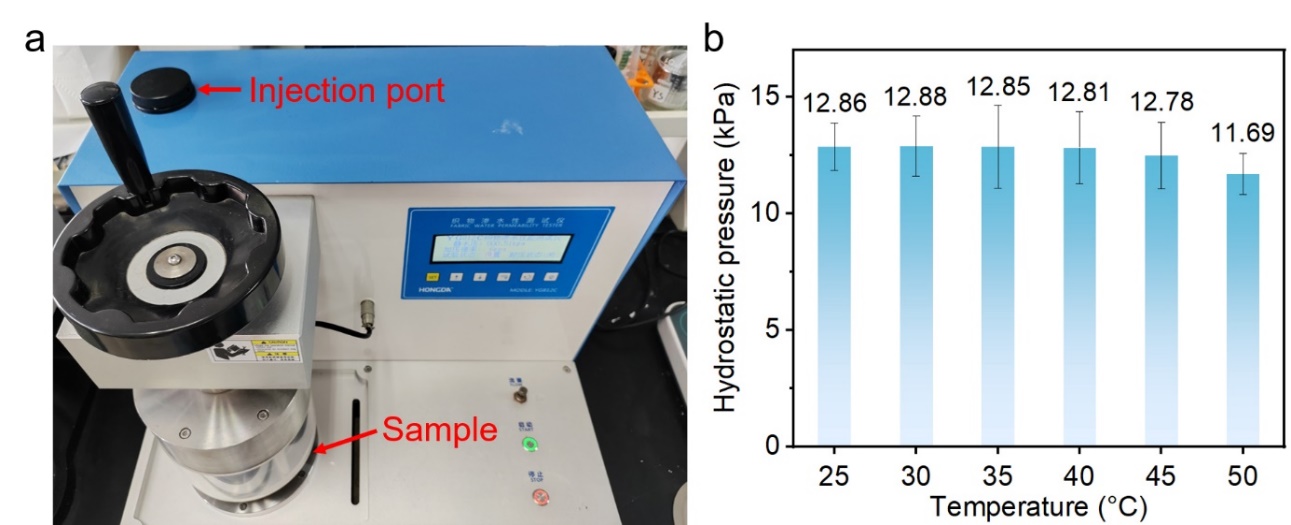


**Fig. S13** **a**) The hydrostatic pressure measurement equipment. **b**) Hydrostatic pressure of HPPT under different temperatures


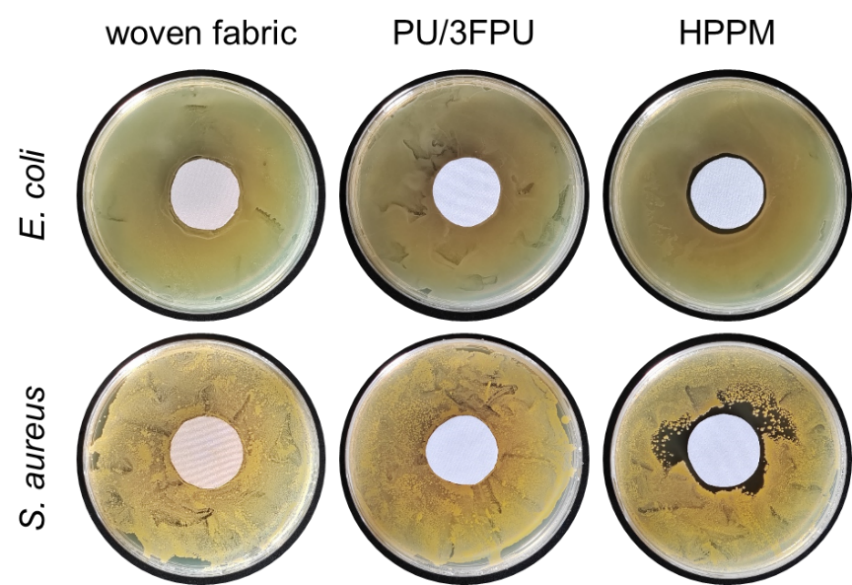


**Fig. S14** Digital photographs of the bacteriostatic effect of woven fabrics, PU/3FPU and HPPT on *E. coli* and *S. aureus* colonies


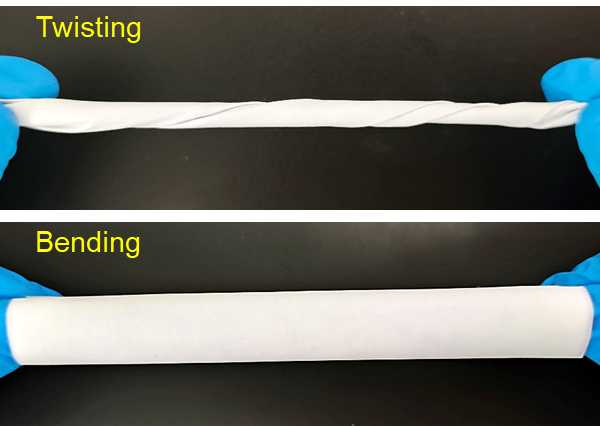


**Fig. S15** Photograph images of twisting and bending tests of HPPT


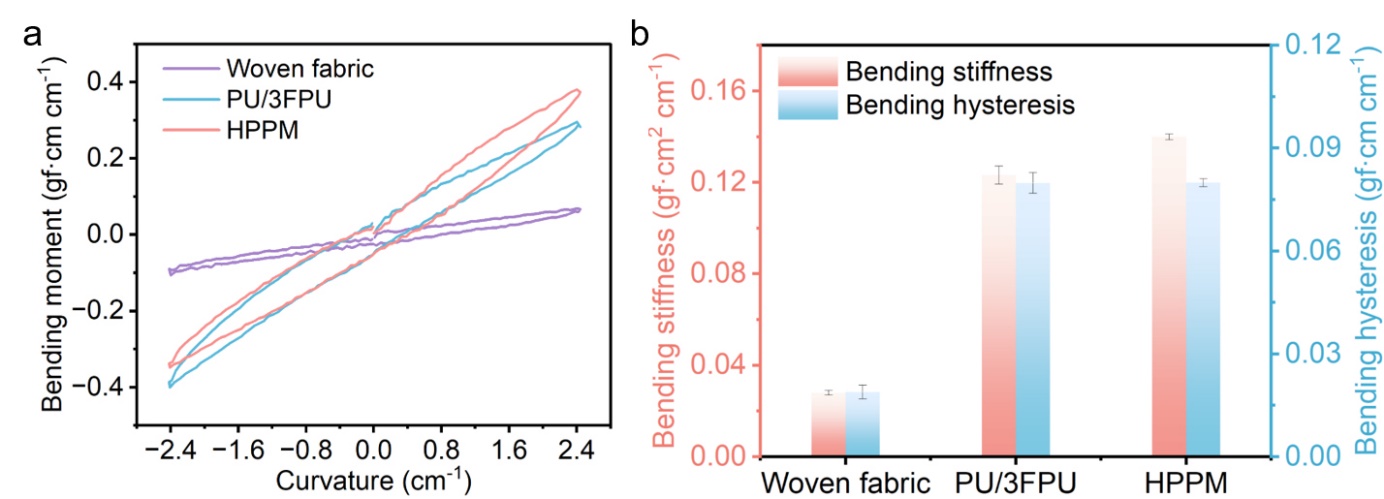


**Fig. S16** Bending properties of woven fabric, PU/3FPU, and HPPT. **a**) The torque-curvature relationship curve. **b**) Bending stiffness and bending hysteresis


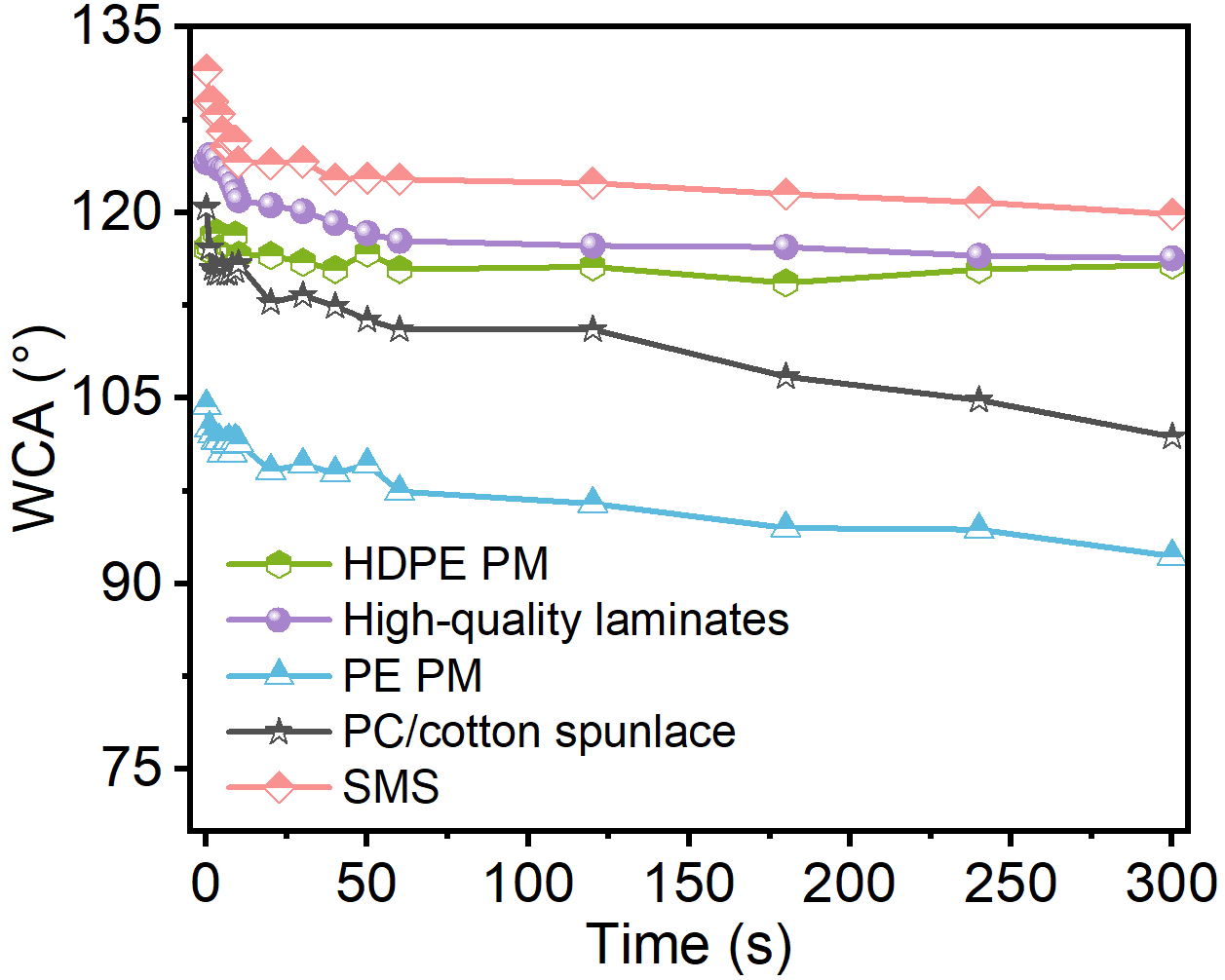


**Fig. S17** The dynamic WCA of five commercial protective materials in 300 s after 1000 abrasion cycles


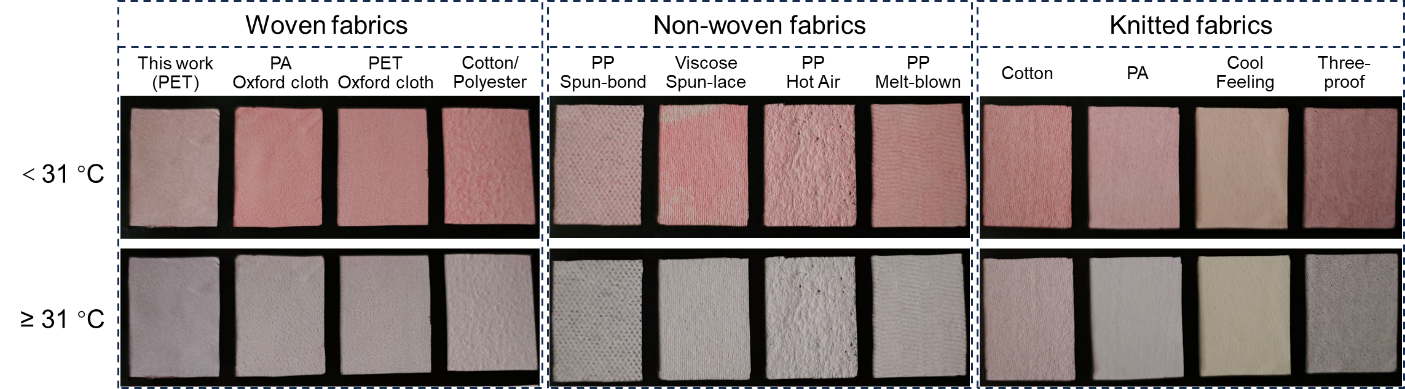


**Fig. S18** Optical images of phase transition membranes before and after a temperature change of 31°C, which are prepared by DNIPT and sprayed technique with the addition of a thermochromic dye to the casting solution on woven fabrics of different materials, non-woven fabrics of fabrication techniques, and knitted fabrics of different materials

(Material parameters:

(1) Woven fabrics: 1-polyester (PET) woven fabric (65 g m^-2^, used in this work), 2-nylon (PA) oxford fabric (210D), 3-PET oxford fabric (210D), 4-PET/cotton poplin (100 g m^-2^);

(2) Non-woven fabrics: 5-polypropylene (PP) spun-bond non-woven (18 g m^-2^), 6-viscose spun-lace non-woven (60 g m^-2^), 7-PP hot-air non-woven (60 g m^-2^), 8- melt-blown non-woven (25 g m^-2^);

(3) Knitted fabrics: 9-cotton sweatband (130 g m^-2^), 10-PA knitted fabric (60 g m^-2^), 11-cool-feeling knitted fabric (45 g m^-2^), 12-black triple-proof knitted fabric (45 g m^-2^))

S2 Supplementary Movies

**Movie S1** The dynamic demonstration of waterproofing and breathability of HPPT

**Movie S2** Dynamic video of water impact on HPPT taken by high-speed camera equipment

S3 Supplementary Experimental Section

**Characterizations.** The morphology and structure of samples and elemental information were acquired using an SU8010 SEM. Pore size distribution of membranes was measured by PMI CFP-1100AI porosimetry. The BET surface area was characterized via the BET surface area analyzer (ASAP 2460). The n-butanol uptake test was used to assess the porosity of membranes. The FTIR spectra were performed using a Nicolet iS10 spectrometer. Phase separation of PU/FPU/CaCl_2_ solutions was observed using a Zeiss Confocal Laser Scanning Microscope (LSM 900).

Kino SL200KS goniometer was applied to measure the dynamic WCA of woven fabric, PU/3FPU membrane, and PU/3FPU@TRG membrane, and the WCA, BCA, OCA, and ECA of PU/3FPU@TRG membrane, respectively. The dynamics of drop impact were captured by an AX200 type 200K-C FASTCAM high-speed camera at 3000 fps. The surface wettability resistance of membranes was characterized by the Y813 Fabric Staining Degree Tester according to ISO 4920. The hydrostatic pressure of fabrics was detected by YG812C water repellent analyzer. The air permeability was assessed by YG461E Automatic Air Permeability Tester. The moisture permeability was tested using YG 601H-Ⅱ computer-type fabric moisture permeability testing apparatus followed by GB/T 12704.1-2009.

The mechanical properties were examined by YG026G-III Electronic Fabric Strength Tester by GB/T 3923.1-2013. The bending properties were examined by the KES-FB2S bending tester (sample dimensions were 100 mm × 100 mm). The abrasion resistance of samples was determined by the Martindale method following GB/T 21196.3-2007 (YG401-6 Martindale abrasion testing apparatus, Shaanxi Changling Textile Mechanical &Electronic Technological CO., Ltd, China). The specimen diameter was 38 mm, the abrasive diameter was 140 mm, the rotational speed of the motion track was 56.3 rpm, and the friction load was 260 g. The laundry-proof property of HPPT was tested according to GB/T 8629-2017 test standard to verify the strong washing resistance of membrane.

Antibacterial Effect Evaluations. Escherichia coli (*E.coli*) (ATCC 25922) and Staphylococcus aureus (*S.aureus*) (ATCC 25923) were used as the bacterial models. The antibacterial effects of the samples were assessed by using zone of inhibition test method. Initially, the bacterial suspension with a concentration of 10^8^ CFU mL^-1^ was cultured. Subsequently, the bacterial solution (100 µL) was evenly spread on the fresh Luria-Bertani (LB) agar plates, and then the samples were placed on the bacteria-coated agar plates. Finally, thethe petri dishes were incubated at 37 ºC for 24 hours.

S4 Supplementary Methods

S4.1 Method for determining casting solution thickness

The casting solution thickness of 250 µm was selected after several parametric preferences. Firstly, we determined the mass fractions of calcium chloride and FPU. Considering the hydrostatic pressure of HPPT needs to meet the usage requirements, we chose 150 µm, 200 µm, 250 µm, 500 µm, and 750 µm, respectively. Moreover, we prepared different thicknesses of highly permeability protective textiles to study the hydrostatic pressure, air permeability and moisture permeability. The results showed that the 250 µm of casting solution thickness could achieve the best synergistic effect of permeability and protection.

S4.2 Design principles

Our highly permeable protective textiles were designed based on two principles: i) to ensure robust liquid repellent and protective properties, the micro-nano network textiles must be assembled small pore sizes and hydrophobic external surface, yet high porosity to rapidly transport air/moisture; ii) to offer preferable dry comfort, the fabric should have large specific surface area to allow sweat absorption and rapid evaporation.

In order to obtain an excellent porous structure, we selected CaCl_2_ as the pore-forming agent for two reasons. Firstly, CaCl_2_ is an extremely hygroscopicity substance. When placed in water, it rapidly dissolve due to its hygroscopic properties and forms hydrated ion. The highly hydrophilic nature of hydrated ion allows them to absorb a large number of water molecules, which accelerates the dissolution of CaCl_2_ into pores during the non-solvent phase separations. Secondly, compared with other salts, CaCl_2_ reacts exothermically with water, which can accelerate the phase separation.

S4.3 Characterization of the phase separation of solutions

We performed the optical microscopy measurement using an LSM 900 Zeiss Confocal Laser Scanning Microscope. Firstly, ~0.1 ml of solution was dropped on a quartz slide, and then the micromorphological evolution process was recorded by the microscope lens. All experimental process parameters, such as light intensity, ambient temperature, and water droplet addition, were kept constant. The confocal microscope shooting speed was 0.2 s/image. The grey levels of the resultant micrographs were quantified using Image J image processing software. The grey levels of these micrographs were proportional to the transmitted light intensity of the solution and ranged from 0 to 255. The relative transmittance (RT) of the solution can be defined by the following equation [S1]:

$RT=\frac{G_{t}}{G_{0}}$ (S1)

where $G_{t}$ and $G_{0}$ are the grey levels of the micrographs of the study solution at time *t* and initial time *t_0_*, respectively.

Since the solidification of a solution in contact with water leads to a decrease in the RT value of the solution, we chose the change in RT as an indicator to characterize the solidification rate of the solution. The deviation of RT indicates the homogeneity of the solution under study, which we consider as the phase separation rate of the various solutions.

Therefore, the RT deviation (RTD) is calculated by [S1]:

$RT_{D}=\frac{G_{tD}}{G_{t}}$ (S2)

where $G_{tD}$ is the deviation of the grey level of the micrograph of the sample at time *t*.

S4.4 Calculation of molecular dynamics

Molecular models of H_2_O, DMF, CaCl_2_, PU, and FPU for the evolution of solution phase transitions were used to simulate. Molecular dynamics (MD) simulations investigate the formation process of micro-nano network structure in the woven fabric-coated wet membrane after immersion in a water coagulation bath. Materials Studio (MS) 2020 was performed for MD simulations. Interestingly, a four-stage change was presented during the phase transition due to the addition of CaCl_2_. The MS software calculates the intermolecular interaction energies of PU/DMF, PU/H_2_O, CaCl_2_/DMF, CaCl_2_/H_2_O, FPU/DMF, FPU/H_2_O, DMF/H_2_O, the solubility parameter of three solution systems with water, mean square displacement and diffusion coefficients of H_2_O, DMF, CaCl_2_, PU, FPU, and the radial distribution function (RDF) between H_2_O and DMF, CaCl_2_, PU, FPU.

In the complete MD simulation, the following steps are generally included: 1) force field determination, which determines the force field parameters according to the simulation system; 2) construction of the initial model according to the experimental system; 3) geometry model optimization and simulation annealing; 4) selection of the appropriate system for molecular dynamics calculation; and 5) data analysis and processing.

The solubility parameter and diffusion coefficient were calculated as follows: Firstly, the models of H_2_O, DMF, CaCl_2_, PU, and FPU were constructed according to the experimental materials respectively, and then the overall assembly was modeled. Subsequently, model optimization was carried out based on Forcite, where Compass was selected for the force field and the temperature was set to 313.15 K. Immediately, canonical ensemble (NVT) was selected for the calculations, and constant-pressure, constant-temperature (NPT) was selected for the calculations to make the model densities closer to the reality. Finally, the corresponding cohesive energy density (CED), MSD, RDF, etc. were selected for analysis.

The cohesive energy density is used to determine the compatibility of polymers. The mixing energy per unit volume is related to the cohesive energy density of the blend and the pure component as follows:^[2]^

$\frac{{\Delta E}_{mix}}{V}=\emptyset_{A}{(\frac{E_{coh}}{V})}_{A}+\emptyset_{B}{(\frac{E_{coh}}{V})}_{B}-{(\frac{E_{coh}}{V})}_{AB}$ (S3)

Where $\emptyset_{A}$ and $\emptyset_{B}$ are the volume fractions of A and B in the blend system. $E_{coh}$ is the cohesion energy of the model and $V$ is the molar volume. The first two can be calculated in the same manner as above by using only one polymer component.

Typically, the mixing energy of a polymer blend is positive, indicating that energy is required for mixing. This is usually expressed in terms of the dimensionless Flory-Huggins parameter $\chi$[S3]:

$\chi=\frac{1}{\emptyset_{A}\emptyset_{B}}\left( \frac{{\Delta E}_{mix}}{k_{B}T} \right)$ (S4)

where the mixing energy is expressed in the volumetric units of Flory-Huggin theory. A common approximation of the Flory-Huggins parameter is [S4]:

$\chi=\frac{\vartheta}{k_{B}T}\left( \delta_{A}-\delta_{B} \right)^{2}$ (S5)

where $\vartheta$ is the reference volume used for $\chi$ and $\delta$ is the solubility parameter [S5]:

$CED=\frac{E_{coh}}{V}$ (S6)

The square root of the cohesive energy density is called the solubility parameter and is calculated as follows [S6, S7]:

$\delta=\sqrt{CED}=\sqrt{\frac{E_{coh}}{V}}$ (S7)

MSD is the sum of the squares of the differences between the absolute values of the moving distance *r_(t)_* and the moving distance *r_(0)_* of all molecules in the casting solution after t times. The MSD of different molecular chains of the PU/3FPU/CaCl_2_ solution system is as follows [S8]:

$MSD=\frac{1}{N}\left\langle\sum_{i=1}^{N} \left| r_{i}\left( t+\Delta t \right)-r_{i}(t) \right|^{2} \right\rangle$ (S8)

where *N* is the number of molecules diffusing, $r_{i}(t+\Delta t)$ is the position of the molecular after moment $\Delta t$, and $r_{i}(t)$ is the position of the molecular at moment *t*.

RDF refers to the variation in particle density as a function of distance and is frequently used to characterize the stacking condition of atoms and the distance between each chemical bond. The RDF curves were calculated as follows [S8]:

$g\left( r \right)=\frac{2V}{N(N-1)}\frac{1}{V_{r}}\sum_{i=1}^{N} \sum_{j==1}^{i-1} \theta(r_{ij}-r)\theta(r+\Delta r-r_{ij})$ (S9)

where $g(r)$ is the RDF, $r_{ij}$ is the distance from molecular *i* to particle *j*, *V* is the total volume of the solutions, and *N* is the total number of molecules in the solutions.

S4.5 Principle of bending properties test

The test was first performed by moving the specimen along a fixed trajectory for forward and reverse curvature, and finally the torque-curvature relationship curve, bending stiffness, and bending hysteresis index were given. The smaller the value of B and 2HB, the better the bending properties [S9].

The bending stiffness B is defined as the bending moment per unit width of the specimen when the curvature of the fabric undergoes a unit change, i.e [S10]:

$B=\frac{\mathrm{dM}}{\mathrm{dK}}$ (S10)

Where, M is the bending moment per unit width specimen, cN·cm cm^-1^; K is the curvature of the specimen, cm^-1^.

The bending hysteresis 2HB reflects the size of the viscosity of the fabric in bending deformation. It is expressed as the difference between the bending moment at K= 0.5 cm^-1^ on the bending deformation curve and the reply curve (KES standard experimental conditions specify).

Supplementary References

1. S. Zhang, H. Liu, N. Tang, S. Zhou, J. Yu et al., Spider-web-inspired PM_0.3_ filters based on self-sustained electrostatic nanostructured networks. Adv. Mater. **32**(29), e2002361 (2020). <https://doi.org/10.1002/adma.202002361>
2. S. Mao, D. Kuldinow, M.P. Haataja, A. Košmrlj, Phase behavior and morphology of multicomponent liquid mixtures. Soft Matter **15**(6), 1297–1311 (2019). <https://doi.org/10.1039/c8sm02045k>
3. T. Tadros, *Encyclopedia of Colloid and Interface Science*, Springer, Berlin, Heidelberg, **2013**. <https://doi.org/10.1007/978-3-642-20665-8>
4. D. Qian, T.C.T. Michaels, T.P.J. Knowles, Analytical solution to the flory-Huggins model. J. Phys. Chem. Lett. **13**(33), 7853–7860 (2022). <https://doi.org/10.1021/acs.jpclett.2c01986>
5. C. Li, A. Strachan, Cohesive energy density and solubility parameter evolution during the curing of thermoset. Polymer **135**, 162–170 (2018). <https://doi.org/10.1016/j.polymer.2017.12.002>
6. J.H. Hildebrand, Solubility of non-electrolytes. Nature **138**(3496), 742 (1936). <https://doi.org/10.1038/138742a0>
7. X. Li, Y. Li, C. Qian, J. Zhao, S. Wang, Molecular dynamics study of the mechanical and tribological properties of graphene oxide-reinforced polyamide 66/nitrile butadiene rubber composites. Appl. Phys. A **129**(4), 276 (2023). <https://doi.org/10.1007/s00339-023-06563-8>
8. H. Tafrishi, S. Sadeghzadeh, R. Ahmadi, F. Molaei, F. Yousefi et al., Investigation of tetracosane thermal transport in presence of graphene and carbon nanotube fillers–– a molecular dynamics study. J. Energy Storage **29**, 101321 (2020). <https://doi.org/10.1016/j.est.2020.101321>
9. S. Sengupta, S. Debnath, A. Sengupta, Fabric bending behaviour testing instrument for technical textiles. Measurement **87**, 205–215 (2016). <https://doi.org/10.1016/j.measurement.2016.03.030>
10. F.M. Wang, *Performance design of apparel fabrics*, Donghua University Press, China **2000**.
